# Improving the anticancer effect of afatinib and microRNA by using lipid polymeric nanoparticles conjugated with dual pH-responsive and targeting peptides

**DOI:** 10.1186/s12951-019-0519-6

**Published:** 2019-08-19

**Authors:** Shu-Ting Hong, Huaching Lin, Chen-Shen Wang, Chih-Hsien Chang, Anya Maan-Yuh Lin, James Chih-Hsin Yang, Yu-Li Lo

**Affiliations:** 1 Institute of Pharmacology, National Yang-Ming University, Taipei, 112 Taiwan; 2 Division of Colorectal Surgery, Cheng Hsin General Hospital, Taipei, Taiwan; 3 Faculty of Pharmacy, National Yang-Ming University, Taipei, 112 Taiwan; 4 Center for Advanced Pharmaceutics and Drug Delivery Research, National Yang-Ming University, Taipei, 112 Taiwan; 5 Department of Medical Research, Taipei Veterans General Hospital, Taipei, 112 Taiwan; 6 Institute of Oncology, National Taiwan University, Taipei, 106 Taiwan

**Keywords:** Afatinib, Epidermal growth factor receptor (EGFR), Nanoparticles, Cell-penetrating peptides, Colorectal cancer, Targeting delivery

## Abstract

**Background:**

The emergence of resistance to chemotherapy or target therapy, tumor metastasis, and systemic toxicity caused by available anticancer drugs hamper the successful colorectal cancer (CRC) treatment. The rise in epidermal growth factor receptor (EGFR; human epidermal growth factor receptor 1; HER1) expression and enhanced phosphorylation of HER2 and HER3 are associated with tumor resistance, metastasis and invasion, thus resulting in poor outcome of anti-CRC therapy. The use of afatinib, a pan-HER inhibitor, is a potential therapeutic approach for resistant CRC. Additionally, miR-139 has been reported to be negatively correlated with chemoresistance, metastasis, and epithelial–mesenchymal transition (EMT) of CRC. Hence, we develop a nanoparticle formulation consisting of a polymer core to carry afatinib or miR-139, which is surrounded by lipids modified with a targeting ligand and a pH-sensitive penetrating peptide to improve the anticancer effect of cargos against CRC cells.

**Results:**

Our findings show that this formulation displays a spherical shape with core/shell structure, homogeneous particle size distribution and negative zeta potential. The prepared formulations demonstrate a pH-sensitive release profile and an enhanced uptake of cargos into human colorectal adenocarcinoma Caco-2 cells in response to the acidic pH. This nanoparticle formulation incorporating afatinib and miR-139 exhibits low toxicity to normal cells but shows a better inhibitory effect on Caco-2 cells than other formulations. Moreover, the encapsulation of afatinib and miR-139 in peptide-modified nanoparticles remarkably induces apoptosis and inhibits migration and resistance of Caco-2 cells via suppression of pan-HER tyrosine kinase/multidrug resistance/metastasis pathways.

**Conclusion:**

This study proposes a multifunctional nanoparticle formulation for targeted modulation of apoptosis/EGFR/HER/EMT/resistance/progression pathways to increase the sensitivity of colon cancer cells to afatinib.

**Electronic supplementary material:**

The online version of this article (10.1186/s12951-019-0519-6) contains supplementary material, which is available to authorized users.

## Background

Colorectal cancer (CRC) is a major cancer type with high incidence and is one of the main causes of cancer-related deaths worldwide [1]. According to other clinical studies, 97% of CRC patients presented with epidermal growth factor receptor (EGFR; human epidermal growth factor receptor 1; HER1) expression, and 80% of them had high EGFR expression associated with tumor metastasis and invasion, thus resulting in poor outcome of common chemotherapeutics [2]. EGFR (ErbB-1) is a 170-kDa transmembrane glycoprotein that comprises intracellular tyrosine-kinase domain and extracellular ligand binding domain, which affect the cell proliferation, survival, and migration [3]. Besides EGFR, three other closely related ErbB/*HER* family of receptor tyrosine kinases have been identified: HER2/neu (ErbB-2), HER3 (ErbB-3), and HER4 (ErbB-4) [4]. The clinical application of monoclonal antibodies (mAbs), e.g., cetuximab or EGFR-tyrosine kinase inhibitors (TKIs), e.g., gefitinib has shown promising results for CRC treatment [5]. However, the emergence of resistance to chemotherapy or target therapy, tumor metastasis, and systemic toxicity caused by available anticancer drugs hamper the successful CRC treatment [6, 7]. One of the major reasons for acquired resistance to anti-EGFR mAb in CRC cells is related to the rise in cell surface EGFR expression and enhanced phosphorylation of HER2 and HER3 [8], indicating that pan-HER is a potential therapeutic target for anti-CRC therapy [9]. Interestingly, the use of pan-HER inhibitors, such as afatinib (Afa), is effective against parental and resistant CRC cells [8], HER2-overexpressed CRC [10], and metastatic CRC [11].

Afatinib (BIBW2992), a second-generation EGFR-TKI, is an orally active and irreversible pan-ErbB inhibitor approved for patients with EGFR-mutated non-small cell lung cancer (NSCLC) [12]. The covalent bond between acrylamide of afatinib and the cysteine residue within the active site of the intracellular tyrosine kinase domain of EGFR, HER2, and HER4 enhances afatinib’s potency against cancer cell growth and induces cancer cell apoptosis [13]. Furthermore, afatinib remarkably enhanced the anticancer activity of adriamycin by inhibiting the P-glycoprotein (P-gp)-mediated multidrug resistance (MDR) in A549T lung cancer cells [14]. Moreover, afatinib is hydrophobic with low bioavailability and thus causes high distribution around the body to result in severe side effects [15]. Although afatinib is potentially effective for the treatment of CRC cells resistant to anti-EGFR mAb [8], the strong and irreversible covalent bond formation between afatinib and EGFR may also appear in normal cells, which may increase afatinib’s uncomfortable adverse events, such as pulmonary, cutaneous, and gastrointestinal (GI) symptoms [16]. Some serious side effects, including grade 3–4 diarrhea, rash or acne, and troubled breathing may occur [17]. Since afatinib is usually administered by oral route, GI symptoms can be even worsened after several doses [18]. Hence, we developed an appropriate delivery system to promote afatinib targeting and penetration into CRC cells to improve its anti-tumor effect and reduce side effects.

MicroRNAs (miRNAs; miRs) are 18–25 nucleotide, non-coding and single-strand RNA molecules, which may target genes critical for regulating proliferation, invasion, metastasis, and cell cycle in different tumor types [19]. Mature miRNA-139 is downregulated in various types of cancer, including CRC [19–21]. Corroborating data have demonstrated that the underexpression of miR-139 is related to aggressive status of invasive colon cancer [21]. Furthermore, samples from patients with CRC show low miR-139 expression, which is associated with high incidence of chemoresistance and metastasis via epithelial–mesenchymal transition (EMT) [19]. Moreover, overexpression of HER2 decreases miR-139 transcription and results in lymph node metastasis in human metastatic gastric cancer [22]. Notably, Bcl2 is a direct target of miR-139 and the anti-tumor efficacy of miR-139 administration is mediated via Bcl2 suppression in CRC [16]. However, the rapid degradation of miR in the systemic circulation and the difficulty in delivery of naked miR-139 into cells prompt the need for the development of suitable systems for miR delivery [19–21].

Lipid–polymer hybrid nanoparticles (LPN) comprise a polymeric core coated with lipid layers as the shell [23]. The surface modification of LPNs by various ligands and cell-penetrating peptide (CPP) may further improve their targeting and enhance their penetration into the tumor site. We chose the CPP H and ligand R to modify on the surface of nanoparticles. CPPs are cationic short peptides that easily penetrate cell membrane [24]. H peptide is a pH-sensitive CPP that usually interacts with cells by forming pores, thus providing the antimicrobial and anticancer activity [25]. This CPP possesses arginine (R) and histidine (H) rich residues, which respond to the acidic pH in tumor microenvironment [26]. In the current study, H peptide was evaluated for its potential pH-sensitive and penetrating characteristics in CRC cells. Peptide R is a ligand binding to neuropilin-1 (NRP-1), a co-receptor for vascular endothelial growth factor [27]. NRP-1 overexpression is associated with CRC angiogenesis and growth [28]. Peptide R was screened from the peptide library with good EGFR-binding and tumor-targeting properties [27, 28]. Peptide R also possesses a prototypic CendR motif responsible for triggering extravasation and tumor penetration [29]. Because afatinib is lipophilic and miR is easily degraded in aqueous phase, we thus designed LPNs composed of polymer polylactic-*co*-glycolic acid (PLGA) as a hydrophobic core to deliver afatinib and/or miR-139, which was surrounded by PEG-lipids as an amphiphilic shell. This shell was further modified with the ligand R and the pH-sensitive CPP H to enhance targeting and penetrating characteristics of these multifunctional nanoparticles in the acidic microenvironment of CRC cells.

## Results

### Physicochemical characterization of afatinib-loaded LPN (Afa/LPN), peptide-modified LPN (Afa/LPN-HR), and miR-139/LPN-HR

**Fig. 1:**
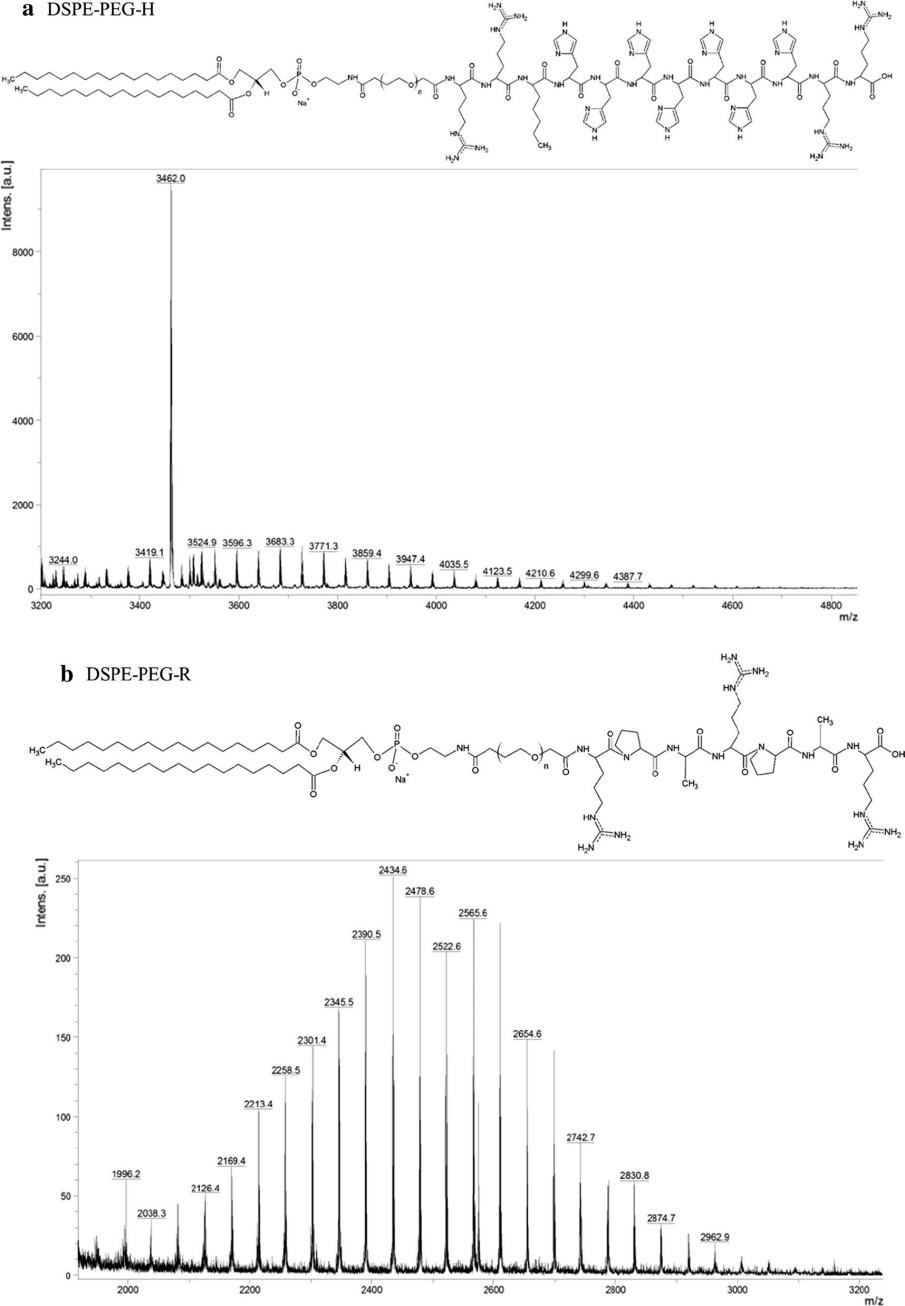
Mass spectra of peptide-conjugated lipids. Mass spectra of **a** DSPE-PEG-H and **b** DSPE-PEG-R. Conjugation of peptide and DSPE-PEG-NHS was characterized by Matrix-Assisted Laser Desorption/Ionization Time-Of-Flight Mass Spectrometry (MALDI TOF MS)

Before obtaining peptide-modified LPN, conjugations of peptide H or R and lipid were performed. Mass spectrometry was used to confirm the conjugation of peptides with lipids. The mass spectra of DSPE-PEG-NHS and H were shown in Additional file 1: Figure S1A and B, respectively. The mass spectrum of DSPE-PEG-H indicated the successful conjugation of DSPE-PEG-NHS and peptide H (Fig. 1a). The mass spectrum of R was displayed in Additional file 1: Figure S1C. The mass spectrum of DSPE-PEG-R also suggested the successful linking of DSPE-PEG-NHS to peptide R (Fig. 1b).

**Fig. 2:**
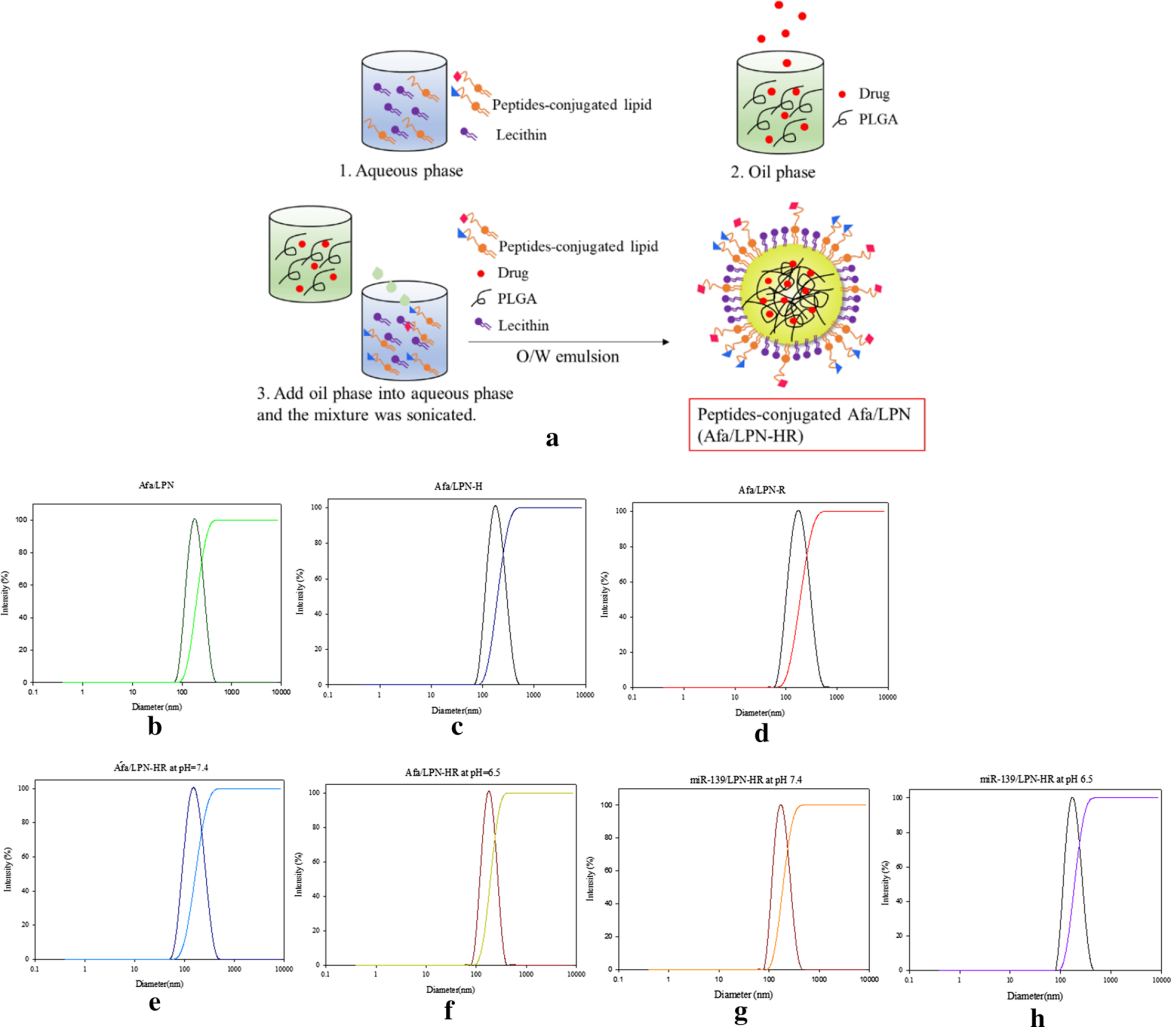
Particle size distribution of afatinib or miR-139 in various LPNs. **a** A schematic diagram for the preparation of peptides-conjugated Afa/LPN. Cumulative size distribution of **b** Afa/LPN; **c** Afa/LPN-H; **d** Afa/LPN-R; **e** Afa/LPN-HR at pH 7.4; **f** Afa/LPN-HR at pH 6.5; **g** miR-139/LPN-HR at pH 7.4; **h** miR-139/LPN-HR at pH 6.5 were measured by Zetasizer. Afa/LPN-HR and miR-139/LPN-HR were diluted at pH 7.4 or 6.5 PBS before measurement

**Fig. 3:**
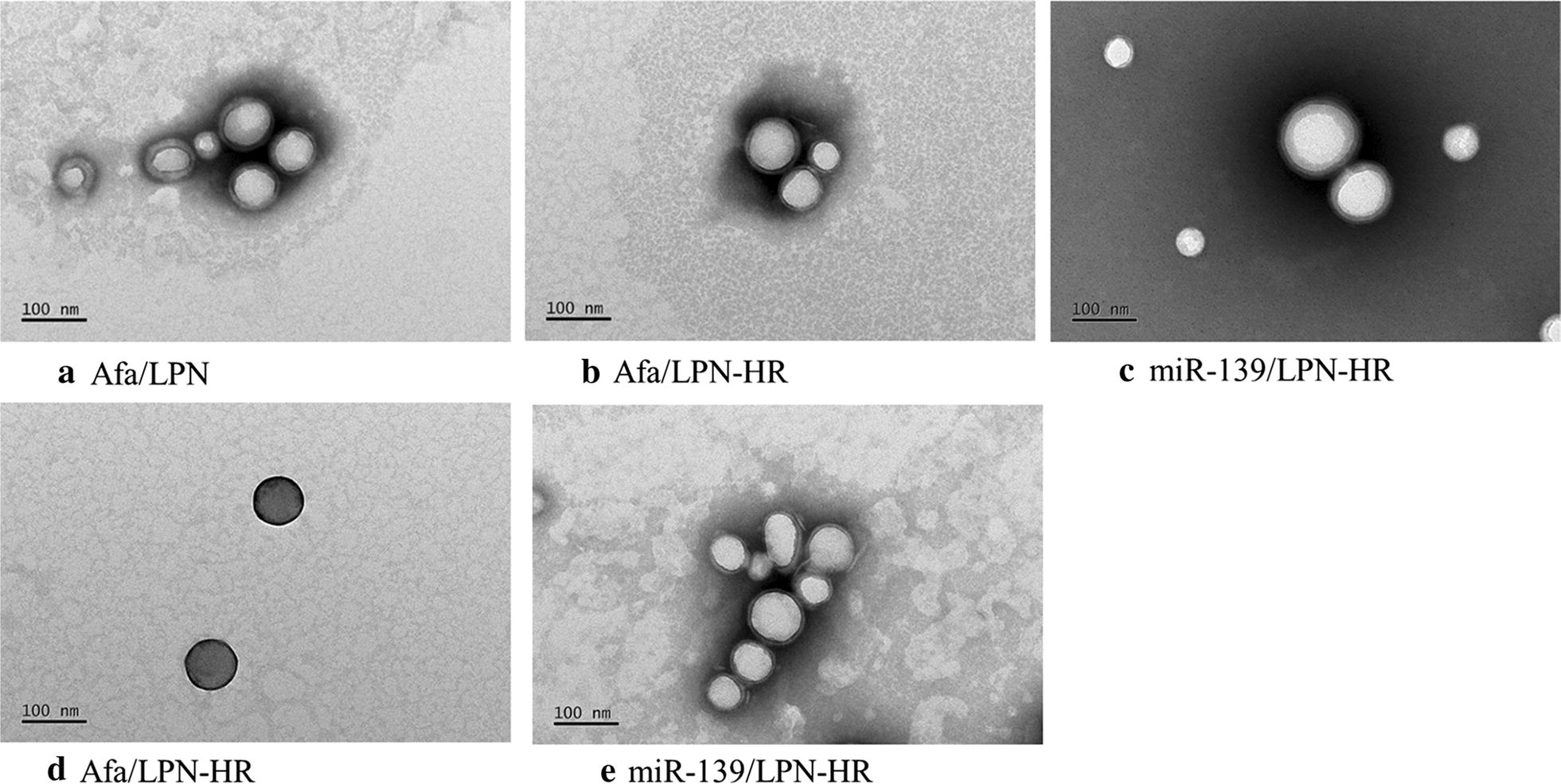
Morphology of afatinib in LPN, LPN-HR or miR-139 in LPN-HR. Transmission electron microscopic (TEM) images of **a** Afa/LPN; **b** Afa/LPN-HR; **c** miR-139/LPN-HR; **d** Afa/LPN-HR (at 4 °C for 28 day-storage); **e** miR-139/LPN-HR (at 4 °C for 28 day-storage). Samples were stained by phosphotungstic acid and morphology of LPNs was observed by TEM. Bar = 100 nm

**Fig. 4:**
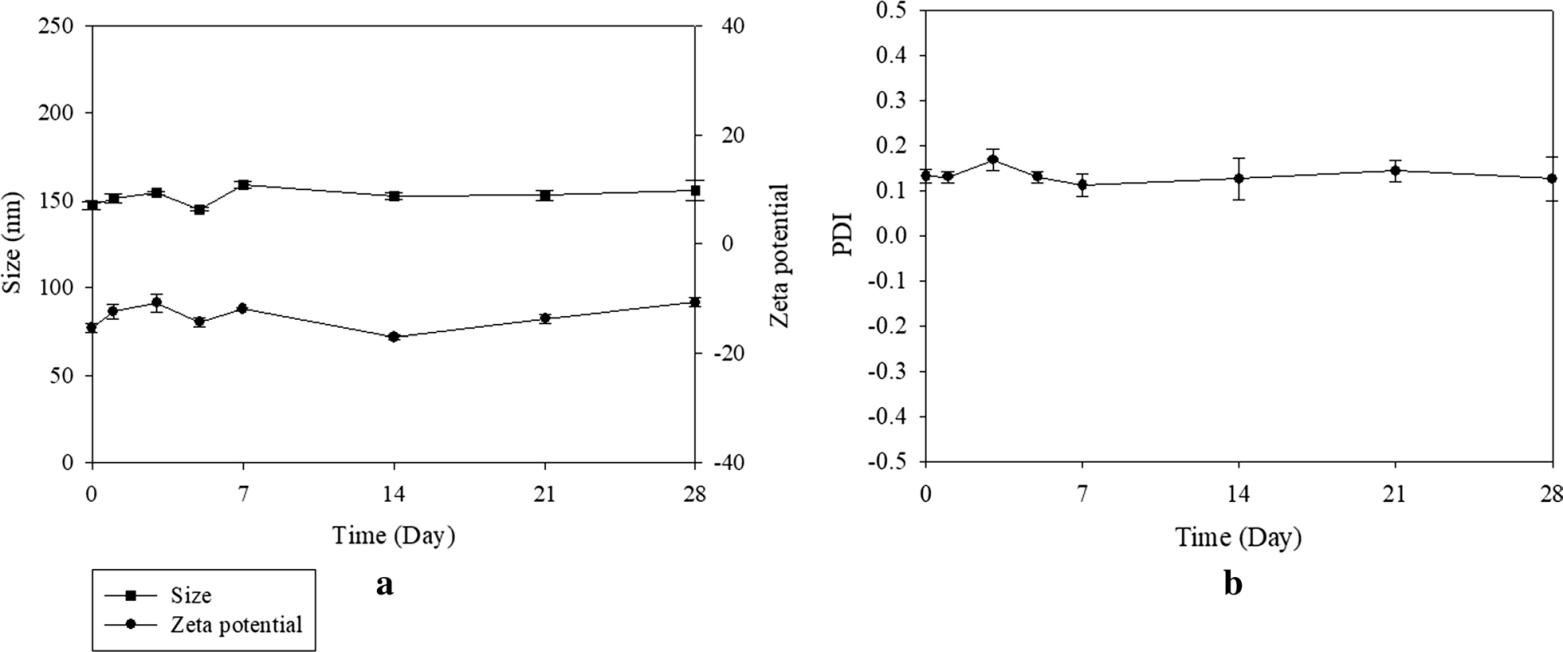
Stability of afatinib in LPN-HR at 4 °C. **a** Size and zeta potential; **b** PDI of Afa/LPN-HR at 4 °C for 28 days. Afa/LPN-HR was stored at 4 °C for 28 days and the size, zeta potential and PDI was measured by Zetasizer. The values are the mean ± standard error (SD). For each group, *n* = 3

The size, zeta potential, polydispersity index (PDI), encapsulation efficiency (EE%) and drug loading capacity (DL%) of various formulations were evaluated (Table 1). The preparation of peptide H and/or R-conjugated Afa/LPN is shown in Fig. 2a. The average size of non-peptide conjugated Afa/LPN and miR-139/LPN as well as peptide-conjugated Afa/LPN and miR-139/LPN was from 141.2 ± 3.1 to 183.8 ± 1.0 nm (Table 1; Fig. 2b, e) and the zeta potential was approximately − 15.2 ± 1.0 to − 5.7 ± 0.9 mV. Given that H is a pH-sensitive peptide and our purpose was to combine H and R for the further modification on LPN, the size and zeta potential of miR-139- and Afa-loaded LPN-HR at pH 7.4 and pH 6.5 were measured. The size of Afa/LPN-HR and miR-139/LPN-HR at pH 7.4 was 147.3 ± 2.3 and 141.2 ± 3.1 nm, which mildly increased to 183.8 ± 1.0 and 175.6 ± 2.6 nm. The zeta potential of Afa/LPN-HR was − 10.2 ± 1.4 mV at pH 7.4, which was increased to − 5.7 ± 0.9 mV at pH 6.5, because peptide H was protonated under acidic environment. The size of Afa/LPN-HR at pH 6.5 was larger than that at pH 7.4. This is at least partially explained by the evidence that the decrease in the value of negative zeta potential at pH 6.5 might reduce the repulsion force among the nanoparticle surfaces. The EE% of Afa/LPN, Afa/LPN-HR, and miR-139/LPN-HR was 87.5 ± 1.7%, 87.3 ± 1.3%, and 86.2 ± 1.8, respectively (Table 1). The DL% of Afa/LPN, Afa/LPN-HR, and miR-139/LPN-HR was 15.4 ± 1.2, 15.3 ± 1.6, and 16.2 ± 0.9, correspondingly (Table 1). The PDI values of all formulations were between 0.1 to 0.2 and distribution of particles size were narrow, indicating that these formulations were homogenous in their distribution (Table 1 and Fig. 2a–f). The shape of Afa/LPN, Afa/LPN-HR, and miR-139/LPN-HR was spherical with a monolayer coated on the surface, as shown by transmission electron microscopic (TEM) images in Fig. 3a–c. In addition, the size, zeta potential, PDI values, and TEM images of Afa/LPN-HR at 4 °C did not change significantly for 28 days, revealing the desirable stability (Figs. 3d, e and 4).

**Table 1:** Characterization of afatinib or miR-139 in various LPNs

Formulations	Particle size (nm)	PDI	Zeta potential (mV)	EE (%)	DL (%)
Afa/LPN	175.5 ± 1.5	0.11 ± 0.04	− 13.1 ± 1.6	87.5 ± 1.7	15.4 ± 1.2
Afa/LPN-H	176.1 ± 2.0	0.12 ± 0.04	− 14.4 ± 1.0	–	
Afa/LPN-R	162.1 ± 1.8	0.13 ± 0.17	− 14.2 ± 1.3	–	
Afa/LPN-HR at pH 7.4	147.3 ± 2.3	0.21 ± 0.01	− 10.2 ± 1.4	87.3 ± 1.3	15.3 ± 1.6
Afa/LPN-HR at pH 6.5	183.8 ± 1.0	0.22 ± 0.01	− 5.7 ± 0.9	–	
miR-139/LPN-HR at pH 7.4	141.2 ± 3.1	0.19 ± 0.11	− 15.2 ± 1.4	86.2 ± 1.8	16.2 ± 0.9
miR-139/LPN-HR at pH 6.5	175.6 ± 2.6	0.22 ± 0.14	− 11.7 ± 1.2	–	

Particle size, PDI and zeta potential of Afa or miR-139 in various LPNs were measured by zetasizer. EE % of Afa in LPN or LPN-HR and miR-139 in LPN-HR was calculated after detection of Afa or miR-139 by HPLC–UV and microplate reader, respectively

*Afa* afatinib, *PDI* polydispersity index, *EE* encapsulation efficiency, *DL* drug loading capacity

### In vitro release of afatinib from LPN or LPN-HR at pH 7.4 or 6.5

**Fig. 5:**
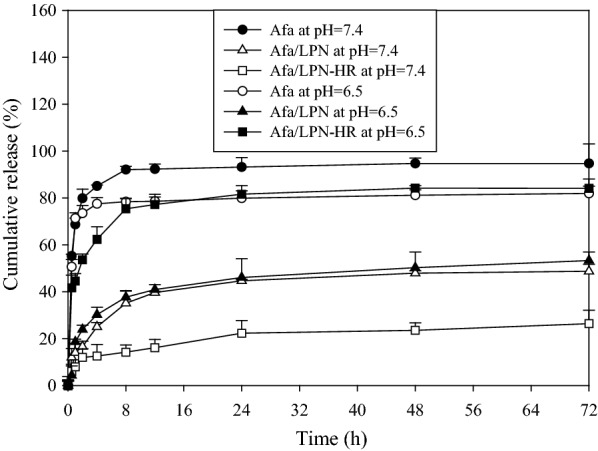
In vitro release of afatinib from LPN or LPN-HR at pH 7.4 or 6.5. The afatinib release profile from different formulations was performed in dialysis bag at pH 7.4 or 6.5. Data are shown as mean ± SD, n = 3

The in vitro release profile showed that 68.70 ± 2.74%, 14.08 ± 3.93%, and 7.99 ± 3.01% of Afa were released from Afa, Afa/LPN, and Afa/LPN-HR, respectively, at pH 7.4 within 1 h (Fig. 5). At 72 h, 94.74 ± 8.36%, 48.73 ± 2.94% and 26.39 ± 5.72% of Afa were released at pH 7.4, correspondingly. Afa showed a burst release profile at 1 h, and both Afa/LPN and Afa/LPN-HR displayed sustained release profiles, especially Afa/LPN-HR, which only released 26.39 ± 5.72% up to 72 h (Fig. 5). By comparison, 18.64 ± 1.11% and 53.33 ± 3.58% of Afa was released from Afa/LPN within 1 h and 72 h at pH 6.5, which was slightly more than that at pH 7.4. Interestingly, 44.54 ± 3.23% and 84.10 ± 0.92% of Afa was released from Afa/LPN-HR at 1 h and 72 h under pH 6.5. Thus, Afa/LPN-HR showed a greater and faster release under acidic environment than that at physiological pH of 7.4 (Fig. 5).

### Toxicity of afatinib in various formulations

**Fig. 6:**
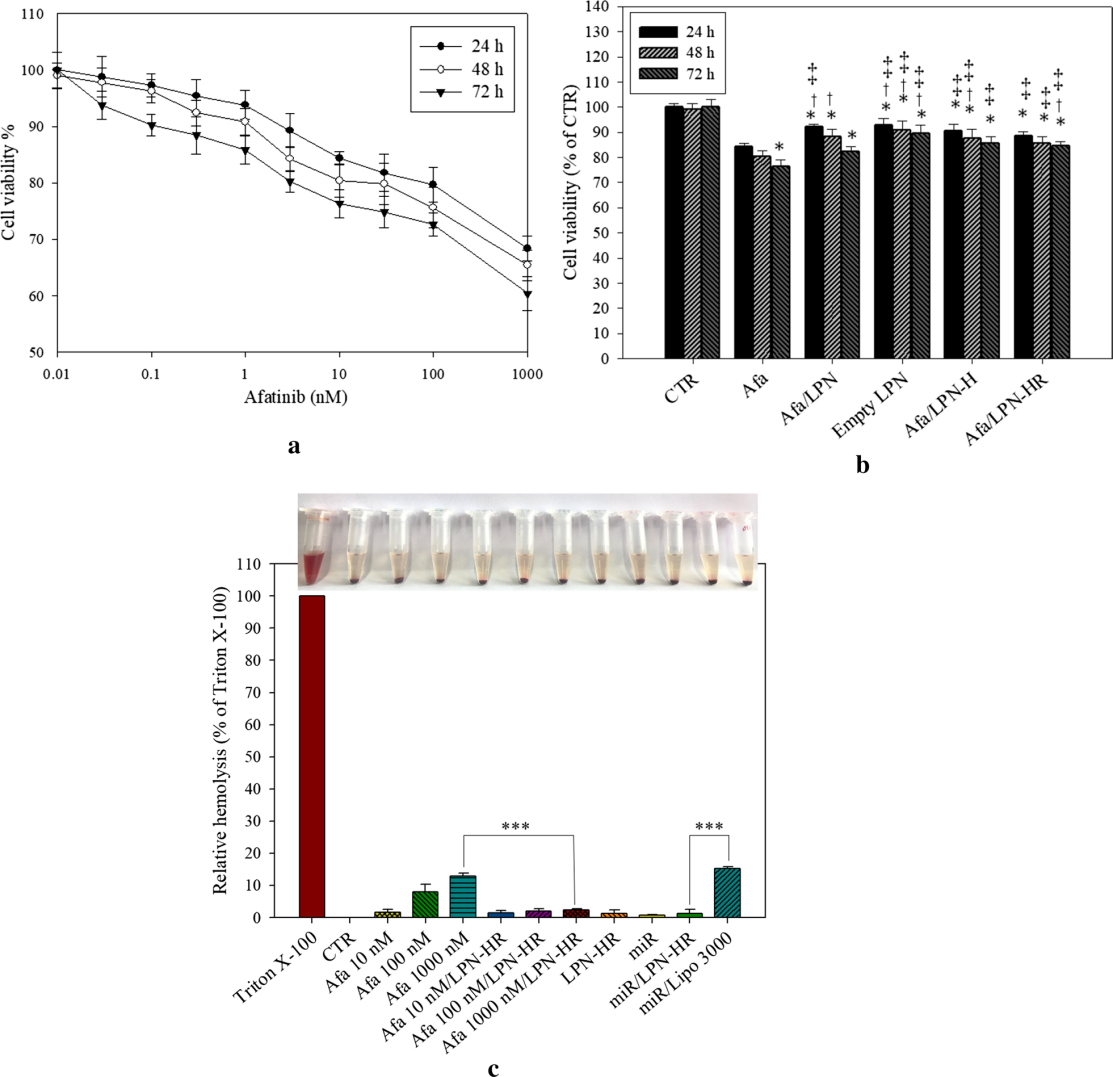
The toxicity of afatinib in various formulations on normal cells. Cytotoxic effect of various formulations on rat intestinal IEC-6 cells or hemolysis effect of these formulations on red blood cells (RBCs). IEC-6 cells were treated with **a** different concentrations of afatinib and **b** afatinib in various LPNs for 24 h, 48 h or 72 h. Cell viability was measured by SRB assay. CTR: cell control; *P < 0.05: compared with Afa for 24 h; ^†^P < 0.05: compared with Afa for 48 h; ^‡^P < 0.05: compared with Afa for 72 h by Student’s t-test. **c** Hemolysis effect was determined by incubating afatinib in various formulations with RBCs at 37 °C for 24 h. Triton-X was used as a positive control. Lipofectamine 3000 (Lipo 3000) is a commercial transfection reagent. Values are the mean ± SD (n = 3)

Rat non-cancerous intestinal epithelial IEC-6 cells were used to evaluate the possible toxicity of these formulations on normal intestinal cells. Various concentrations of afatinib were treated on IEC-6 cells for 24 h, 48 h or 72 h. When the concentration of Afa was increased to 10 nM, the cell viability of IEC-6 was 84.38 ± 1.19% after 24-h treatment (Fig. 6a). When the treatment time was increased to 72 h, the cell viability of IEC-6 was reduced to 76.39 ± 2.49%. 10 nM of Afa was encapsulated in different formulations and the cytotoxicity on IEC-6 cells was compared with Afa treatment for 24 h, 48 h or 72 h, respectively (Fig. 6b). The result indicated that the cell viability of empty LPN carrier (without Afa) was 92.73 ± 2.70% after 24 h-incubation, and all LPN formulations with Afa encapsulation reduced cytotoxicity of Afa against IEC-6 cells after 24 h, 48, or 72 h incubation, correspondingly (Fig. 6b).

Given that the proposed peptide-conjugated nanoparticles are most possibly administrated by IV injection, the potential toxicity of these formulations on blood cells should be tested. Red blood cells from Sprague–Dawley (SD) rats were used for hemolysis test. Different concentrations of afatinib in various formulations were added into blood and incubated at 37 °C for 24 h. Triton-X was used as a positive control of hemolysis. As shown in Fig. 6c, increasing concentrations of Afa to 1 μM caused mild hemolysis (12.85 ± 0.99%). LPN-HR carrier displayed negligible hemolysis (1.23 ± 1.18%) and LPN-HR loaded with different Afa concentrations did not induce toxicity to RBC (< 3% hemolysis). Incorporation of miR-139 in LPN-HR did not result in obvious hemolysis (Fig. 6c). However, miR-139 in Lipofectamine 3000, a commercial transfection reagent, caused more significant hemolysis compared to that of miR-139 in LPN-HR (*P* < 0.001).

### Cytotoxic effect of afatinib and afatinib in various formulations on Caco-2 cells

**Fig. 7:**
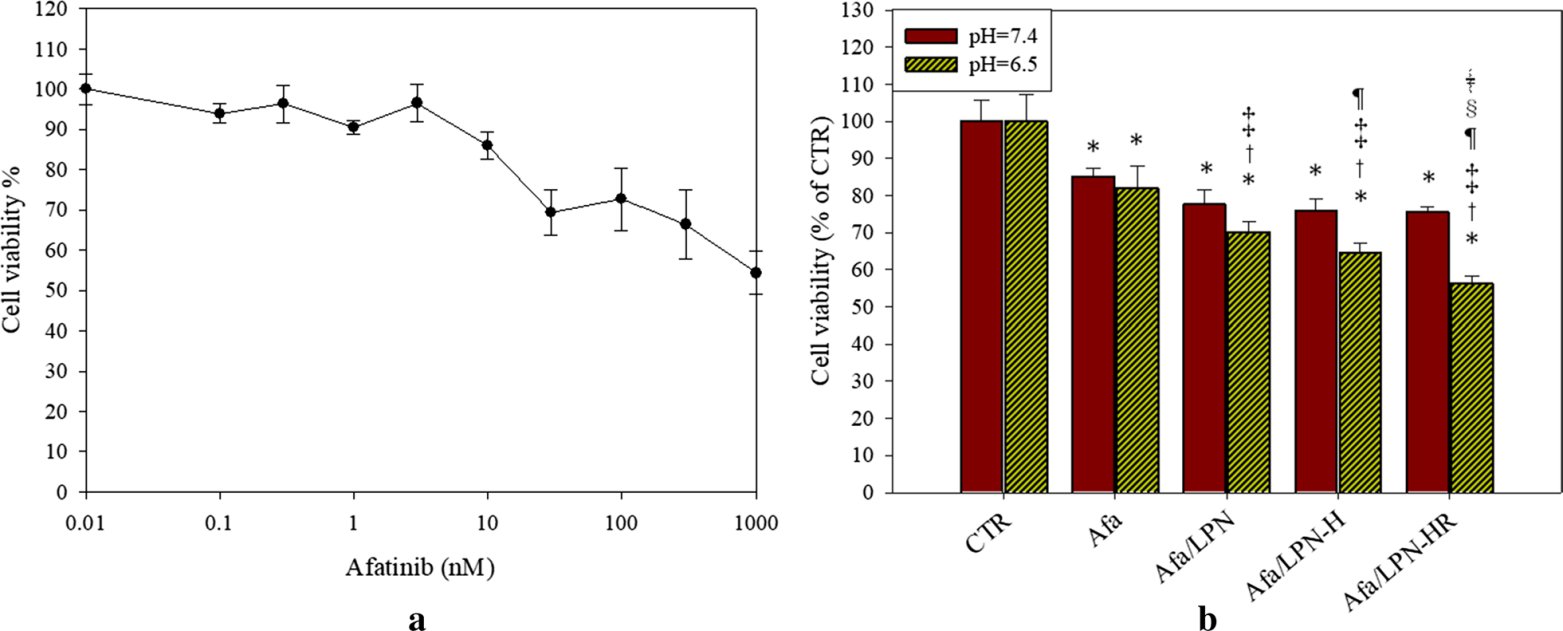
Cytotoxic effect of afatinib in various formulations on Caco-2 cells. Caco-2 cells were treated with **a** different concentration of afatinib and **b** 10 nM of afatinib in various LPNs at pH 7.4 or 6.5 for 72 h. Cell viability was measured by SRB assay. Values are the mean ± SD (n = 3). *P < 0.05: compared with CTR at pH 7.4, ^†^P < 0.05: compared with Afa at pH 7.4, ^‡^P < 0.05: compared with Afa/LPN at pH 7.4, ^¶^P < 0.05: compared with Afa/LPN-H at pH 7.4, ^§^P < 0.05: compared with Afa/LPN at pH 6.5, ^ǂ^< 0.05: compared with Afa/LPN-H at pH 6.5 by Student’s t-test

The cell viability of afatinib in various formulations was evaluated on colon cancer Caco-2 cells for 72 h. 10 nM of Afa decreased the cell viability of Caco-2 cells to 85% and was selected as the Afa concentration for encapsulation in LPN formulations in the following experiments (Fig. 7a). Afa concentration to cause approximately 15% cytotoxicity to Caco-2 cells was selected to verify if Afa/LPN formulations modified with CPP H and/or ligand R might display more cytotoxicity against Caco-2 cells. Results showed that Caco-2 cell viability treated with Afa and Afa/LPN-HR was reduced from 85.10% to 75.44% (relative % of the control group; Fig. 7b). We further investigated the cell viability of Afa in various formulations at pH 6.5 (Fig. 7b) and found that Afa/LPN inhibited more cell growth compared to free Afa at pH 6.5. Afa/LPN-H decreased cell viability to 64.65 ± 2.55% with significant difference compared to that of Afa (81.95 ± 5.88%; *P* < 0.05) and Afa/LPN (70.21 ± 2.91%) at pH 6.5. Dual peptide-modified Afa/LPN-HR further inhibited cell viability to 56.21 ± 2.03% at pH 6.5 compared to 75.44 ± 1.44% at pH 7.4, which showed much better inhibitory effect at acidic pH environment (Fig. 7b).

**Fig. 8:**
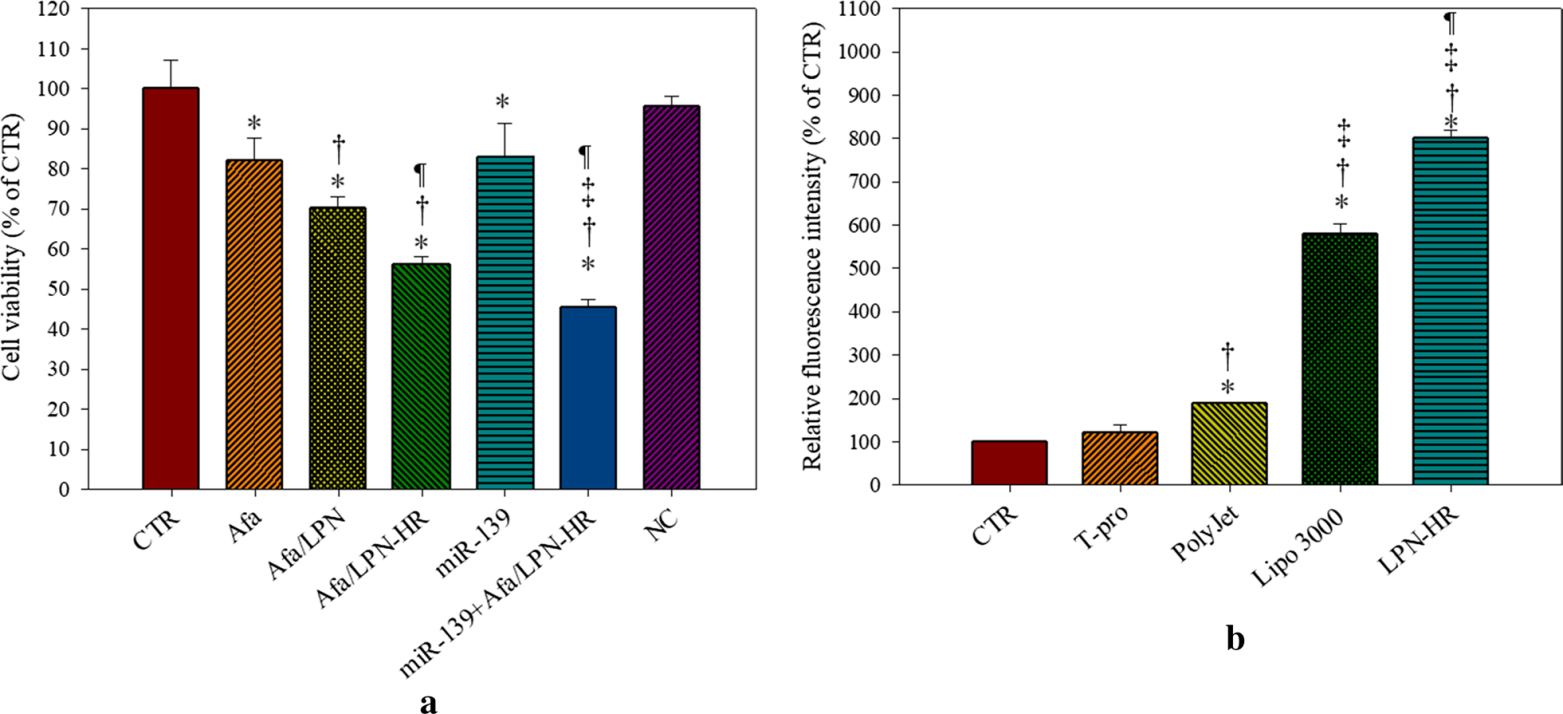
**a** Cytotoxic effect of miR-139 combined with afatinib in various formulations and **b** transfection efficiency of FAM-miR139/LPN-HR and other transfection reagents in Caco-2 cells. Values are the mean ± SD (n = 3). For **a** *P < 0.05: compared with CTR, ^†^P < 0.05: compared with Afa, ^‡^P < 0.05: compared with Afa/LPN-HR, ^¶^P < 0.05: compared with miR-139 by Student’s t-test. NC was as a negative control. For **b** *P < 0.05: compared with CTR, ^†^P < 0.05: compared with T-pro, ^‡^P < 0.05: compared with PolyJet, ^¶^P < 0.05: compared with Lipo 3000 by Student’s t-test

After confirming the cytotoxicity of Afa/LPN-HR, we further evaluated whether the combined treatment with miR-139/LPN-HR enhanced the cytotoxicity of Afa/LPN-HR. Pretreatment of Caco-2 cells with miR-139/LPN-HR for 1 h and then co-treatment with Afa/LPN-HR for 72 h inhibited more cell growth compared to Afa, Afa/LPN-HR, and miR-139/LPN-HR alone groups (Fig. 8a). Pretreatment of miR-139/LPN-HR for 1 h, and then treatment with Afa/LPN-HR for 72 h showed the best inhibitory effect against Caco-2 cell growth (Fig. 8a).

Transfection efficiency of fluorescein amidite (FAM)-labeled miR-139 by LPN-HR and commercial transfection reagents was measured using a flow cytometer. Results showed that the fluorescence intensity of FAM-miR-139 loaded in LPN was eightfold of the control group (Fig. 8b). FAM-miR-139/LPN also demonstrated higher transfection % than those of commercial transfection reagents, including Lipofectamine 3000, T-pro, and PolyJet (Fig. 8b). LPN-HR displayed the greatest transfection efficiency compared to other groups. Thus, LPN-HR demonstrated a great potential to enhance cellular uptake of miR-139 and showed better transfection efficiency than other commercial transfection reagents used in the present study (Fig. 8b).

### Uptake of coumarin-6 (C-6) or FAM-miR139-loaded LPNs into Caco-2 cells

**Fig. 9:**
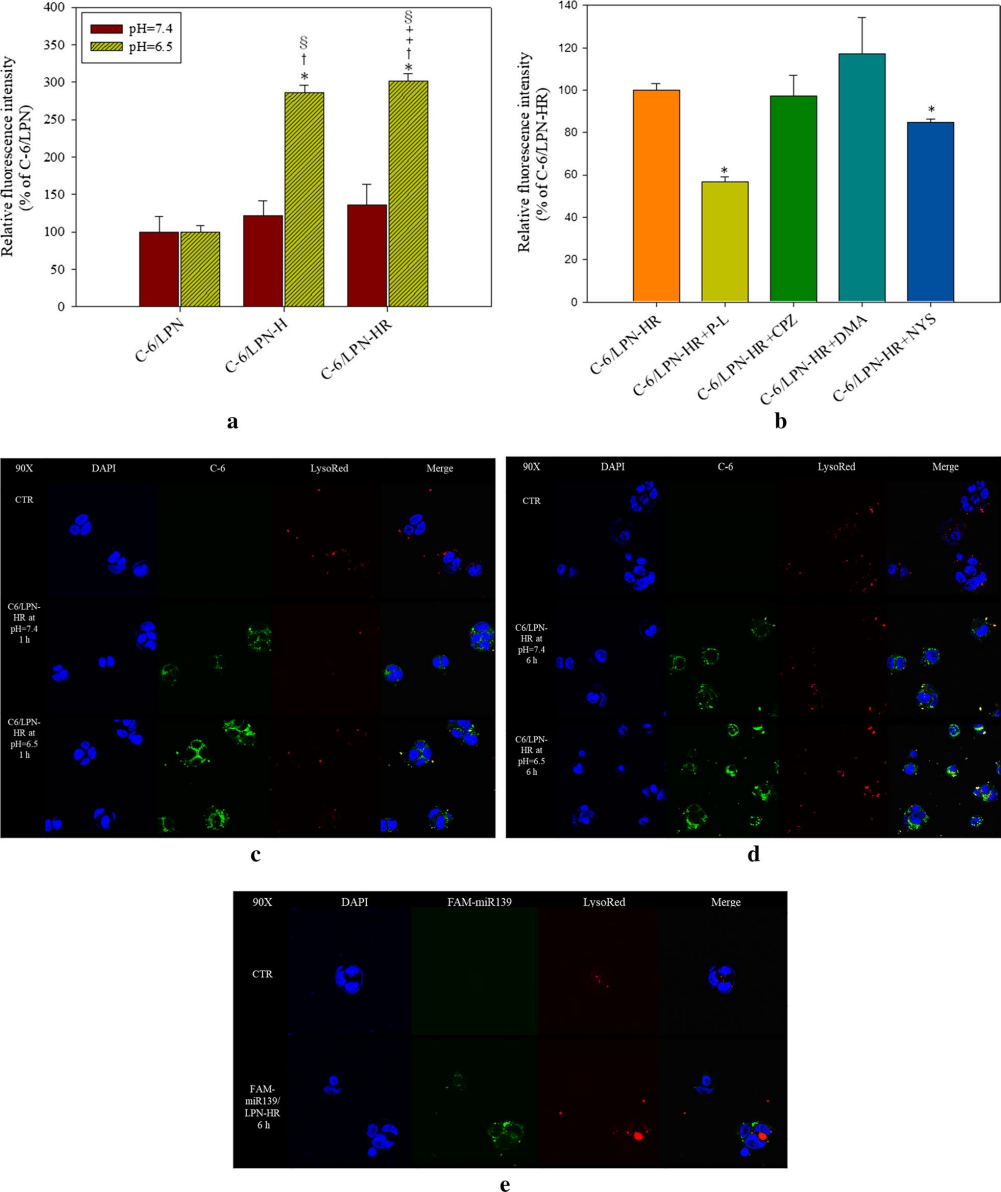
The uptake of coumarin-6-(C-6) or FAM-miR139/LPN-HR into Caco-2 cells. **a**, **b** Caco-2 cells were treated with **a** coumarin-6 in various LPNs at pH 7.4 and 6.5 for 6 h or **b** pretreated with different endocytosis inhibitors for 1 h and then treated with C-6/LPN-HR at pH 6.5 for 6 h. Various endocytosis inhibitors include P-L (adsorptive endocytosis inhibitor), CPZ (clathrin-mediated endocytosis inhibitor), DMA (macropinocytosis inhibitor), and NYS (caveolae-mediated endocytosis inhibitor). Cellular uptake was measured by detecting the relative fluorescence intensity (green fluorescence) using a flow cytometer. **a** *P < 0.05: compared with C-6/LPN at pH 6.5, ^†^P < 0.05: compared with C-6/LPN-H at pH 7.4, ^‡^P < 0.05: compared with C-6/LPN-H at pH 6.5, and ^§^P < 0.05: compared with C-6/LPN-HR at pH 7.4. **b** *P < 0.05: compared with C-6/LPN-HR. Values are the mean ± SD (n = 3). **c**–**e** The localization of C-6/LPN-HR at pH 7.4 or 6.5 in Caco-2 cells at **c** 1 h and **d** 6 h; **e** Caco-2 cells were treated with FAM-miR139/LPN-HR for 6 h and observed by confocal laser scanning microscope (CLSM). Green: C-6 or FAM-miR139; Red: LysoRed; Blue: DAPI. Magnification: ×90

We further evaluated the cellular uptake mechanism(s) of Afa/LPN-HR in Caco-2 cells. Coumarin-6 (C-6) was used as a green fluorescent marker of afatinib for incorporation into LPNs. The cellular C-6 levels after delivering by different LPN formulations at pH 7.4 and 6.5 were quantified by flow cytometry. As shown in Fig. 9a, there was no significant difference between the C-6 uptake level by LPN-H, LPN-R, or LPN-HR at pH 7.4 (all *P* > 0.05). However, cellular C-6/LPN-H was 2.36-fold at pH 6.5 compared to that at pH 7.4. Particularly, C-6/LPN-HR at pH 6.5 showed the highest C-6 fluorescence than all other groups (all *P* < 0.05; Fig. 9a).

To further investigate which pathway was involved in transporting C-6/LPN-HR into the cells, cells were pretreated with various endocytosis inhibitors for 1 h and treated with C-6/LPN-HR for 6 h. The fluorescence of C-6/LPN-HR pretreated with poly-lysine (C-6/LPN-HR + P-L) and nystatin (C-6/LPN-HR + NYS) was significantly diminished compared with that of C-6/LPN-HR without endocytosis inhibitors. The result demonstrated that C-6/LPN-HR might be transported by adsorptive- and caveolae-mediated pathway (Fig. 9b).

The intracellular localization of FAM-miR139/LPN-HR and C-6/LPN-HR in Caco-2 cells were observed by confocal laser scanning microscopy (CLSM). After various treatments, cells were stained with 4′,6-diamidino-2-phenylindole (DAPI; blue color) and LysoTracker Red (LysoRed; red color) to localize nucleus and lysosomes, correspondingly. The localization of C-6/LPN-HR in Caco-2 cells was observed after 1- and 6-h incubation at pH 7.4 and 6.5. At both time points, green fluorescence, including green spots of C-6/LPN-HR appeared mainly in the cytoplasm, but slightly co-localized with lysosomes (red) after 6-h uptake (Fig. 9c, d). Moreover, compared to pH 7.4, cellular uptake of C-6/LPN-HR was higher at pH 6.5 and more green dots of C-6 were observed in cells at 6 h (Fig. 9d). In addition, green fluorescence of FAM-miR139/LPN-HR was not co-localized with red fluorescence of LysoTracker, indicating that miR-139/LPN-HR was internalized into cytoplasm after transfection in Caco-2 cells for 6 h (Fig. 9e).

### Effect of Afa/LPN-HR combined with miR-139/LPN-HR on apoptosis and EGFR/pan-HER in Caco-2 cells

**Fig. 10:**
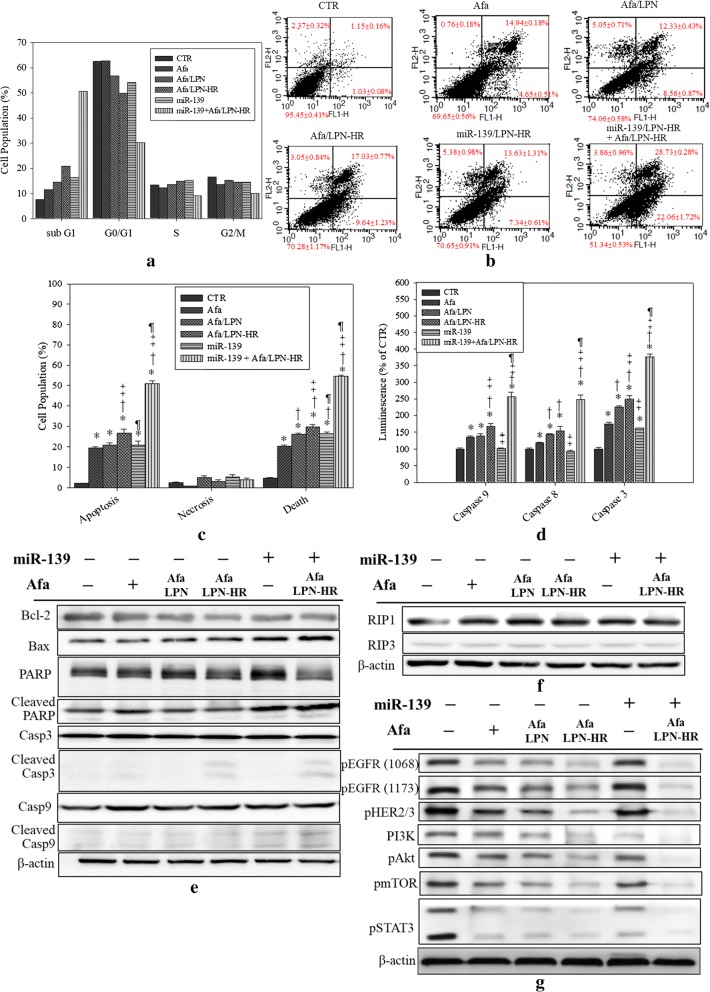
Effect of treatment with various formulations of Afa and/or miR-139 on different death mechanisms in Caco-2 cells. **a** Cell cycle assay was determined by PI staining and the fluorescence was measured by flow cytometry. **b** FACS analysis of different cell populations was performed by Annexin V and PI staining. **c** The relative percentage of the apoptotic, necrotic and dead cell population; **d** Caspase activity was measured by detecting the relative luminescence using a microplate reader. For **c** and **d**, the values are the mean ± SD (*n* = 3). *P < 0.05: compared with CTR. ^†^P < 0.05: compared with Afa. ^‡^P < 0.05: compared with Afa/LPN. ^¶^P < 0.05: compared with Afa/LPN-HR. **e**–**g** Protein expressions of **e** apoptosis, including Bax, Bcl-2, caspase 9, caspase 3 and PARP, **f** necroptosis such as RIP1 and 3, as well as **g** EGFR/HER and PI3 K/Akt/mTOR/STAT3 pathways were detected by western blot after the above treatment in Caco-2 cells

After confirming that the treatment of Afa/LPN-HR combined with miR-139/LPN-HR enhanced the cytotoxicity against Caco-2 cells, we further assessed the cell cycle by propidium iodide (PI) staining. Sub-G1 phase represented apoptotic cell populations. Afa/LPN-HR increased sub-G1 phase to 20.98 ± 0.09% (compared to free Afa 11.49 ± 0.64%), which enhanced the apoptosis of Caco-2 cells. miR-139 alone caused 16.37 ± 0.26% of apoptosis, which was similar to that caused by Afa/LPN (14.59 ± 0.95%). The co-treatment of miR-139/LPN-HR and Afa/LPN-HR remarkably escalated apoptosis population to 50.67 ± 0.88% in Caco-2 cells, which supported the effective cell growth inhibition by this formulation (Fig. 10a).

We also used Annexin V-FITC/PI double-staining to quantify cell apoptosis and/or necrosis (%). We found that the apoptosis percentage (the sum of early and late apoptotic cells) and death percentage (the sum of apoptotic and necrotic cells; Fig. 10b, c) triggered by Afa/LPN-HR were considerably greater than those induced by Afa or Afa/LPN (all *P* < 0.05). Specifically, the combined treatment of miR-139/LPN-HR and Afa/LPN-HR induced more apoptotic and dead cells compared to other groups (Fig. 10b, c). Furthermore, necrotic population almost remained at similar low % in all the formulations (all *P* > 0.05; Fig. 10b, c).

Caspase activity assay was also used to confirm apoptotic effect of each formulation. Caspase 9, 8, and 3 activity levels were performed by adding Caspase-Glo^®^ kit to cells and detect the luminescence signal. Compared to the control group, the luminescence of caspase 9, 8 and 3 in the groups of Afa, Afa/LPN, Afa/LPN-HR, and/or miR-139/LPN-HR were significantly increased, especially miR-139/LPN-HR combined with Afa/LPN-HR, which activated caspase 9, 8 and 3 activities the most (Fig. 10d).

Apoptotic protein expression of Caco-2 cells after treatments for 72 h was evaluated by western blot. First, the expression levels of pro-apoptotic proteins, such as Bax, cleaved poly (ADP-ribose) polymerase (PARP), caspase-3 and -9, were increasingly upregulated by the treatments of Afa/LPN-HR and/or miR-139/LPN-HR. By contrast, Bcl-2, an anti-apoptotic protein, was downregulated by the combined treatment of miR-139/LPN-HR and Afa/LPN-HR (Fig. 10e). The kinases receptor-interacting protein 1 (RIP1) and RIP3, two necroptosis regulators, were not obviously changed in all groups (Fig. 10f). Additionally, co-treatment of miR-139/LPN-HR and Afa/LPN-HR substantially suppressed the protein expression of phosphorylated forms of EGFR (phospho-EGFR; Tyr1068 and 1173), phospho-HER2/3 (Tyr1221/1222), PI3K, as well as phospho-forms of Akt, mTOR, and STAT3 (Fig. 10g).

### Effect of various Afa- and/or miR-139 formulations on β-catenin-, EMT-, MDR- and Rac1/KRAS/MAPK-related pathways in Caco-2 cells

**Fig. 11:**
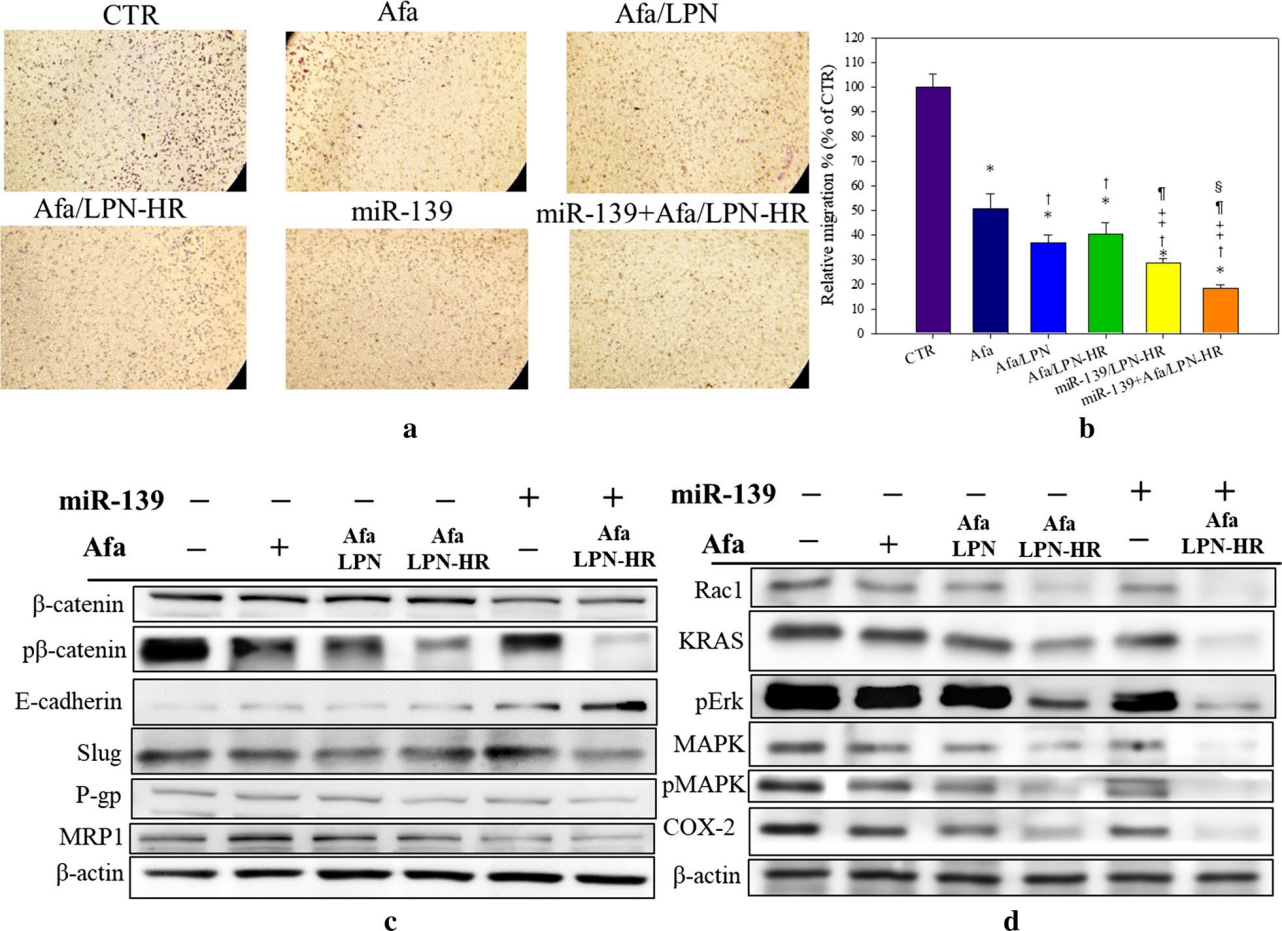
Effect of various treatments on migration, MDR and other pathways in Caco-2 cells. **a** Migration assay was evaluated on Transwell inserts. After different treatments, the cells were stained with 0.2% crystal violet and observed by microscope. Purple dots were the migrated cells across the Transwell inserts. **b** Relative percentages of migrated cells compared to CTR. Data are shown as mean ± SD, n = 3. *P < 0.05: compared with CTR, ^†^P < 0.05: compared with Afa, ^‡^P < 0.05: compared with Afa/LPN, ^¶^P < 0.05: compared with Afa/LPN-HR, and ^§^P < 0.05: compared with miR-139/LPN-HR. **c**, **d** Protein expressions of **c** EMT/MDR and **d** Rac1/Ras/Erk/MAPK/COX-2 pathways were evaluated after various treatments on Caco-2 cells for 72 h by western blot. The experiments were performed in triplicate with the similar results

Cell migration assay was performed on Transwell inserts. There were numerous migrated cells remained on the opposite side of insert membrane in the control group (Fig. 11a). Treatment with free Afa noticeably inhibited approximately 50% cell migration (Fig. 11b). The migration ability of Caco-2 cells was mildly further inhibited by treatment of Afa/LPN or Afa/LPN-HR (Fig. 11b). Most importantly, the combined treatment of miR-139- and Afa/LPN-HR further reduced the cell migration % across the membrane to 18.43 ± 1.29% (Fig. 11b).

The protein expression of β-catenin-, EMT-, MDR- and Rac1/KRAS/MAPK-associated pathways was evaluated by western blot on Caco-2 cells after treatment with various formulations. Combined treatment with miR-139/LPN-HR and Afa/LPN-HR considerably inhibited the expressions of β-catenin/phosphor-β-catenin and Slug as well as markedly escalated the expression of E-cadherin compared with the other treatment groups (Fig. 11c). Interestingly, this co-treatment also remarkably suppressed the expression of P-gp and MRP1 (Fig. 11c). Furthermore, this combined treatment also markedly inhibited the protein expressions of Rac1, KRAS, Erk, MAPK, and COX-2 compared with the other treatment groups (Fig. 11d).

## Discussion

Among patients with CRC, some of them have poor survival based on the expression of EGFR associated with tumor growth. EGFR activation or mutation and/or HER2/3 phosphorylation contribute to tumor progression and resistance [30]. Afatinib is an oral irreversible EGFR-TKI with broad spectrum activity against EGFR and HER2/3/4 [31]. A previous investigation indicated that afatinib, a pan-HER inhibitor, is effective against colon cancer cells resistant to anti-EGFR therapy [8]. We proposed that the combined treatment of afatinib and miR for regulating multiple anticancer signaling pathways may effectively suppress CRC. Among various miR, miR-139 was frequently found to be downregulated in CRC [21]. The expression of miR-139 is lower in CRC cells than in normal tissues [21, 32]. In addition, the reduced expression of miR-139 is correlated with CRC progression and metastasis in human colon cancer [32]. We specially selected human colorectal adenocarcinoma Caco-2 cells as the model CRC cell line in the present study. Given that Caco-2 cells overexpress EGFR, HER2/3, MDR1, and MDR-associated protein (MRP)1/2 [33], Caco-2 can represent a resistant CRC cell line with broad spectrum resistance. Caco-2 cells also express NRP1, which is a non-tyrosine kinase receptor associated with tumor migration and survival [34]. In the current study, we chose R peptide to selectively target NRP1 expressed on CRC cells. Additionally, we used pH-responsive H peptide for enhancing LPN to change in tumor acidic microenvironment. This delivery system of LPN combines the advantages of using biodegradable polymer (PLGA) and biocompatible phospholipids as carrier constituents to achieve small particle size, functional surface, high drug loading, excellent stability, and specific release of cargos [35, 36].

The peptide–lipid conjugation formed amide bonds, in which NHS on the lipid–PEG terminal reacted with primary amine groups of peptide H or R under basic environment and the NHS group left after completing the reaction. The mass spectra of DSPE-PEG-H or DSPE-PEG-R indicated that the proposed peptide–lipid conjugates were successfully synthesized (Fig. 1a, b). The size of prepared LPN formulations ranged from 141.2 ± 3.1 to 183.8 ± 1.0 nm, which could be efficiently internalized into cells (Table 1). PEG linked on the phospholipids stabilized LPN and reduced adsorption of proteins, such as albumin, in systemic circulation [37]. The DL% of Afa/LPN, Afa/LPN-HR, and miR-139/LPN-HR were 15.4 ± 1.2, 15.3 ± 1.6, and 16.2 ± 0.9, correspondingly (Table 1), which might affect the therapeutic effect of these nanoparticle formulations. Furthermore, it has been pointed out that nanomedicines usually display the disadvantage of limited drug-loading (mostly less than 10%) due to more carrier materials [38]. Recently, the nanoparticles with drug-loading capacity more than 10% have drawn growing interest [38, 39]. However, these high drug-loading nanomedicines are prepared using inert porous material as carrier such as mesoporous silica nanoparticles and mesoporous carbon nanoparticles [39]. In the present study, since afatinib is a lipophilic drug, the drug loading capacity into hydrophobic PLGA core was thus escalated into a higher than 10% level. Surprisingly, the loading of miR-139 into LPN-HR was also more than 10% in the current study. Consistently, recent evidences have shown that PLGA is an excellent delivery material for nucleic acid-based cargos, including plasmid DNA, siRNA, and miRNA [40, 41].

The zeta potential of Afa/LPN-HR at pH 6.5 was less anionic than that at pH 7.4 due to protonation of histidine (pKa ~ 6) residues in peptide H under acidic environment to bear more positive charges [42].

The pH sensitivity of LPN-HR could be clearly verified by the in vitro release profile of afatinib from different formulations. The LPN formulation was composed of PLGA core, which was a biodegradable polymer and well known for its sustained release ability [43]. The release of Afa/LPN at pH 7.4 for 72 h was approximately 48.73 ± 2.94%, which exhibited a sustained release mode. However, the conjugation of peptides H and R on LPN reduced the release of afatinib from LPN-HR (26.39 ± 5.72% for 72 h at pH 7.4). This may be caused by the intensified hydrophobic interaction between histidine groups of peptide H and the PLGA core, which made the drug stay at the core longer at pH 7.4 [44]. When pH was reduced to 6.5, histidine became hydrophilic after protonation of its imidazole ring, which decreased its interaction with PLGA core and caused faster release (84.10 ± 0. 92% for 72 h at pH 6) (Fig. 5). Recently, the similar tumor-specific and pH-responsive peptide was also used to prepare theranostic liposomes for incorporating paclitaxel [45]. They also confirmed the pH-sensitive characteristics and antitumor efficacy of these theranostic liposomes in triple negative breast cancer MDA-MB-231 cells in vitro and in vivo [45].

Although nanoparticles are suitable for cancer delivery, the toxicity of nanoparticles on normal cells may be a concern. Furthermore, the administrative route of these nanoparticle formulations are mostly via IV injection [46]. According to a previous study, hemolysis percentage of formulations should be below 10% [47]. We found that encapsulation of Afa in LPN or LPN-HR diminished its toxicity on IEC-6 cells and reduced the hemolysis % to less than 10% on RBC (Fig. 6). Moreover, LPN, LPN-H or LPN-HR increased cytotoxicity against cancer cells at the acidic environment (Fig. 7). Modification of LPN by pH-sensitive H peptide and NRP1 targeting ligand R showed enhanced the cytotoxicity on NRP1-expressed Caco-2 cells at pH 6.5 due to protonation of histidine in H peptide under acid environment (Fig. 7b). Similar result was found in the cellular uptake of C-6/LPN-HR, which was significantly increased at pH 6.5 (Fig. 9). Interestingly, Cryer et al. designed afatinib-gold nanoconjugates to reduce the side effects and enhance efficacy of afatinib for the possible application in the treatment of NSCLC [15]. This nanoconjugate formulation was demonstrated to maintain the viability of human alveolar epithelial type I-like cells, suggesting a good biocompatibility of this delivery system to healthy lung epithelium [15].

Our current findings of cytotoxicity, cell cycle, and annexin V/PI staining all showed that Afa/LPN-HR combined with miR-139 further increased Caco-2 cell death (mostly via apoptosis) than that of Afa/LPN-HR alone (Figs. 8 and 10). This increase might be related to the direct targeting of miR-139 to anti-apoptotic regulator Bcl-2, which is involved in drug resistance and progression of cancer cells [48]. Another study also demonstrated that miR-139 directly targeted to Bcl-2 or Mcl-1 to regulate downstream pathway of apoptosis and cell death [19]. Accordingly, the expression of Bcl-2 was considerably downregulated by the combined treatment of miR-139 and Afa/LPN-HR (Fig. 10e). Furthermore, Bax and cleaved caspase 9, 3 and PARP were activated after co-treatment of miR-139- and Afa/LPN-HR (Fig. 10d, e). Caspase 9 is the initiator of intrinsic apoptosis pathway to trigger caspase 3 activation, thus provoking the mitochondria-mediated apoptosis [49]. Caspase 3 is also responsible for PARP cleavage, which is associated with DNA fragmentation during cell death [50, 51]. Consistently, Coelho et al. found that afatinib linked to PEGylated gold nanoparticles increased uptake and cytotoxicity of afatinib against pancreatic and lung cancer cells [52].

**Fig. 12:**
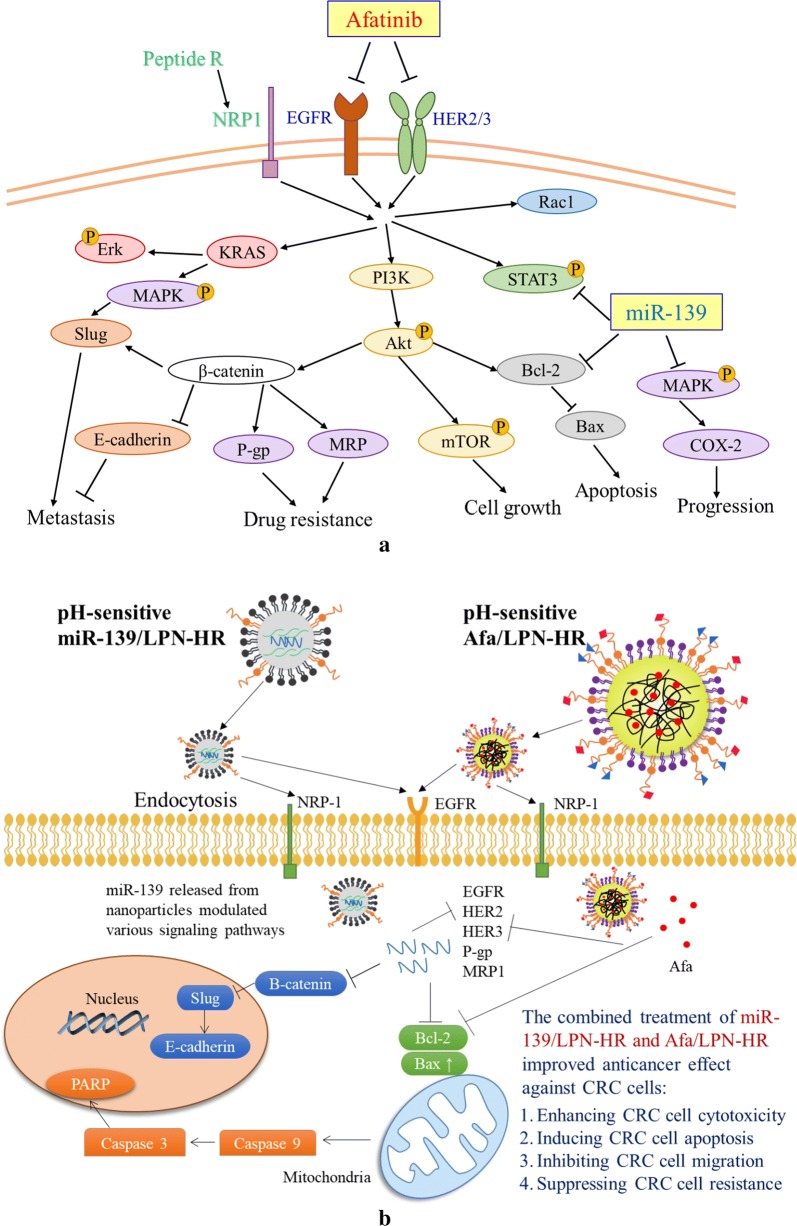
**a** Afatinib- and/or miR139-loaded LPN-HR inhibited EGFR/HR signaling via suppression of Ras-, Akt-, Rac1-, and STAT3-mediated pathways. **b** Schematic diagram of peptide H- and peptide R-modified lipid polymeric nanoparticles incorporating afatinib and miR-139 to intensify anticancer activity against CRC cells

Interestingly, a previous study revealed that the expression levels of EMT proteins, such as vimentin and ZEB1, were also decreased in miR-139 transfected cancer cells [19]. Our result supported this statement by showing that the combined treatment of miR-139- and/or Afa-loaded LPN-HR diminished the protein expression of β-catenin and Slug, but escalated the expression of E-cadherin (Fig. 11c). Additionally, this co-treatment also suppressed EGFR, KRAS and its downstream phospho-MAPK expression, thus reducing Slug expression (Fig. 11d). We thus verified in this study that co-treatment of miR-139- and Afa-loaded LPN-HR inhibited EMT via dual suppression of EGFR and β-catenin signaling pathways (Figs. 10g, 11c, d). Consistently, this formulation reduced the relative migration % of Caco-2 cells (Fig. 11a, b). Furthermore, HER2 and/or HER3 are upregulated in many tumor cells, such as CRC and gastric cancer cells [9, 22]; overexpression of these EGFR-family proteins has concordance with resistance, metastasis, and poor prognosis of various cancers [11, 22]. Moreover, the direct binding of HER2 to CD44 inhibited transcription of miR-139, thus causing lymph node metastasis in human metastatic gastric cancer [22]. MiR-139 is usually downregulated in HER2-overexpressed breast and gastric cancer [53]. Remarkably, our finding suggested that co-treatment of miR-139- and Afa-incorporated LPN-HR considerably suppressed expression of phosphorylated proteins of EGFR, HER2/3, Akt, mTOR, and STAT3 (Fig. 10g), which supported the inhibitory effect of this formulation on resistant CRC cells accompanying with acquired activation of EGFR/pan-HER family and the related STAT3, PI3K, and KRAS signaling pathways. Hence, we provide proof-of-concept evidences to support that pan-HER inhibitor such as afatinib might effectively inhibit EGFR/HER/Akt/MAPK pathways, which are activated in resistant CRC, as indicated by Khelwatty et al. [8]. Besides, co-treatment of miR-139/LPN-HR and Afa/LPN-HR also significantly reduced the protein expression of Rac1 and KRAS and the associated phospho-Erk, phospho-MAPK, and COX-2 (Fig. 11d), thereby notably weakening cancer cell growth and progression. Most importantly, this formulation noticeably diminished the protein expressions of P-gp and MRP1 (Fig. 11c), which further confirmed the excellent repressive effect of this formulation on MDR transporter-mediated resistance in CRC cells. As supported by the findings in Figs. 10 and 11, we thus suggest that co-treatment of miR-139/LPN-HR and Afa/LPN-HR might modulate multiple signaling pathways to suppress drug resistance, cancer cell growth, progression and metastasis as well as induce apoptosis in CRC cells (Fig. 12a). A proposed schematic of peptide H- and peptide R-modified lipid polymeric nanoparticles incorporating afatinib and miR-139 to intensify anticancer effect against CRC cells is shown in Fig. 12b.

## Conclusions

Collectively, we develop a multifunctional nanoparticle formulation of afatinib and miR-139 with targeting, penetrating, and pH-responsive characteristics for simultaneous modulation of EGFR/HER/Ras/Akt/Rac1/STAT3/MAPK/EMT/Bcl-2 pathways to increase the sensitivity of colon cancer cells to afatinib. This formulation also demonstrates the advantage of low toxicity to normal cells. To our best knowledge, this is an innovative investigation linking the pH-responsive targeting nanoparticles to ErbB-tyrosine kinase signaling inhibition, metastasis and multidrug resistance reversal along with programmed cell death induction in colon cancer cells.

## Methods

### Materials

Peptide H and R were purchased from Kelowna Biotech (Taipei, Taiwan) at > 95% purity. miR-139 and FAM-miR139 were purchased from Genepharma (Shanghai, China). DSPE-PEG2000-NHS was obtained from Nanocs Inc. (Boston, MA, USA). Afatinib and PLGA were purchased from Sigma-Aldrich (St. Louis, MO, USA). Monoclonal antibodies (Mab) of Bax, Bcl-2, β-catenin, caspase 9, caspase 3, PARP, RIP1, RIP3 and E-cadherin were purchased from Cell Signaling Technology (Beverly, MA, USA). Slug Mab was from Abcam (Cambridge, UK). β-actin goat anti-mouse and rabbit IgG antibody was purchased from Millipore (Billerica, MA, USA). All cell culture medium and reagents were bought from Promega (Madison, WI, USA), Invitrogen (Carlsbad, CA, USA), Gibco BRL (Grand Island, NY, USA), or Hyclone (Logan, UT, USA). All other chemical reagents were obtained from either Merck (Darmstadt, Germany) or Sigma-Aldrich (St. Louis, MO, USA).

### Preparation of afatinib-loaded LPN-HR or miR-139-loaded LPN-HR

Peptide-conjugated lipids were firstly sythesized by mixing peptide H or R and DSPE-PEG-NHS at a molar ratio 1:1 for 24 h at room temperature. The resulting compounds were dialyzed against water by dialysis bag (3.5–5 kDa MWCO membrane, Spectrum Inc., CA, USA). The purified peptide-conjugated lipids were obtained by freeze-drying and characterized by mass.

Afatinib/LPN-HR were prepared by an o/w emulsion method. 3 mg/ml of mPEG-DSPE or peptide-conjugated lipid and lecithin were prepared separately in ethanol/water solution. PLGA (50:50 LA:GA; MW 35,000–45,000) was prepared at a concentration of 2 mg/ml in acetone. For example, to prepare 10 nM Afa/LPN-HR, 2 µl of 20 μM afatinib in DMSO was added into PLGA stock solution. mPEG-DSPE or peptide-conjugated lipid and lecithin were added into PBS buffer to prepare aqueous dispersion. Then, afatinib/PLGA solution was carefully added dropwise into the aqueous lipid medium. The resulting dispersion was sonicated for 10 min using a bath sonicator. The organic solvent was removed by evaporation. The nanoparticles were filtered by 0.45 μm pore size filter and stored at 4 °C.

Preparation of miR-139/LPN-HR was similar to that of afatinib-loaded nanoparticles. For example, to prepare 100 nM miR-139/LPN-HR, 20 µl of 20 μM miR-139 was added into PLGA solution. The miR-139/PLGA solution was then added into the aqueous lipid dispersion. The following steps were the same as the preparation of afatinib-loaded nanoparticles.

### Characterization of afatinib or miR-139 in various LPN formulations: size distribution, zeta potential, EE%, DL% and TEM images

Size distribution and zeta potential of nanoparticles were measured by Zetasizer Nano ZS90 (Malvern Instruments Ltd., Malvern, Worcestershire, UK) at 25 °C with a scattering angle of 90.0°. PDI was calculated by Zetasizer family software v7.11. Records were analyzed from three individual measurements.

The morphology of nanoparticles was observed by TEM (JEM-1400Plus, Japan). The samples were dropped on the grid for 1 min, and the excess of solution was drawn off from the edge with a filter paper. After that, the specimens were stained by 1% phosphotungstic acid for 45 s. Excess solution was blotted with a filter paper, and the samples were dried in the air at room temperature.

A dispersion of Afa- or miR-139 containing nanoparticles was centrifuged at 15,000 rpm and 4 °C through an ultracentrifuge filter (Amicon^®^, MW: 10 kDa). The filtrate was collected and analyzed by HPLC–UV detector and microplate reader (TECAN Sunrise, Männedorf, Schweiz), respectively. Each sample was detected in triplicate. EE% or DL% of Afa or miR-139 in LPN or LPN-HR were calculated by the following formula.

1 $$ {\text{EE}}\% = \left[ {\left( {{\text{W}}_{\text{e}} {-}{\text{W}}_{\text{f}} } \right)/{\text{W}}_{\text{e}} } \right] \times 100\% $$

2 $$ {\text{DL}}\% = \left[ {\left( {{\text{W}}_{\text{e}} {-}{\text{W}}_{\text{f}} } \right)/{\text{W}}_{\text{t}} } \right] \times 100\% $$

where W_e_ is the weight of added Afa or miR, W_f_ is the weight of Afa or miR in the filtrate, and W_t_ is the total nanoparticle weight.

The HPLC system is composed of a PM1110 pump (Hitachi, Tokyo, Japan), an autosampler (Primaide 1210), a C18 column (Phenomenex), and a L2420 UV–VIS detector (Hitachi). The mobile phase was composed of water, acetonitrile, and methanol (55:25:20 v/v). The solution was degassed by a sonicator before detection. The flow rate was 1.0 ml/min, and UV detection was performed at wavelength 254 nm.

### In vitro drug release of afatinib from LPN

The release of afatinib from nanoparticles was performed by dialysis method. 1 ml of afatinib formulation was added into a dialysis bag (3.5–5 kDa MWCO membrane, Spectrum, Inc., Rancho Dominguez, CA, USA). The dialysis bag was placed into a 37.5 ml pH 7.4 or 6.5 PBS with stirring at 400 rpm, and the temperature was maintained at 37 °C. At the indicated time, an aliquot of 0.1 ml sample was obtained from dialyzer, and the same volume of PBS was replaced into dialyzer to maintain the original volume of solution. The nanoparticles were broken by acetonitrile with sonication. The afatinib concentration in each sample was detected by HPLC. The cumulative release of afatinib was then calculated.

### Hemolysis test

Blood (1.5 ml) was collected from SD rat by cardiac puncturing with heparin. RBCs were separated by centrifugation of whole blood at 1500 rpm for 10 min at 4 °C. This step was continued until the supernatant was clear. The supernatant containing plasma and platelets was discarded. The RBC pellet was suspended in 12 ml PBS and divided into 12 tubes (1 ml per tube). Different formulations were added to the tubes, which were then incubated at 37 °C for 24 h. Triton X was used as a positive control. Each group of samples was mixed with Drabkin’s reagent (Sigma, St. Louis, MO, USA). Hemoglobin was oxidized to cyanmethemoglobin after the reaction with Drabkin’s reagent (Sigma). The concentration of cyanmethemoglobin was measured at 540 nm by using Tecan Infinite microplate reader (Männedorf, Switzerland).

### Cell culture

Rat small intestinal epithelial IEC-6 cells and human colorectal adenocarcinoma Caco-2 cells were grown in Dulbecco’s modified Eagle’s medium (DMEM) supplemented with 10% fetal bovine serum (FBS), penicillin/streptomycin and sodium bicarbonate. All these cells were maintained in an incubator with 5% CO_2_ at 37 °C.

### Cytotoxicity of various Afa/LPN or miR-139/LPN-HR on Caco-2 or IEC-6 cells

Caco-2 and IEC-6 cells were seeded at the density of 1 × 10^4^ cells/well and 4 × 10^3^ cells/well in 96-well plates. Different concentrations of afatinib and formulations were added to the medium and incubated for 72 h for Caco-2 cells and 24, 48, or 72 h for IEC-6 cells. After treatments, the cultured medium was removed, and the cytotoxicity was determined by sulforhodamine B assay. The absorbance was measured at 540 nm by Tecan microplate reader.

### Transfection efficiency of miR-139/LPN-HR

Caco-2 cells were seeded at the density of 1 × 10^5^ cells/well in a 24-well plate and cultured for 48 h. FAM-miR-139/LPN-HR and other formulations were added into the plate and incubated for 24 h. After treatments, the cells were collected and centrifuged at 300*g* at 4 °C and then suspended in cold PBS. The samples were analyzed by FACSCalibur flow cytometer. Fluorescence was detected through a FL1 filter for FAM-miR139. Data acquirement and computation were calculated by BD Biosciences software. Each treatment was performed in triplicate.

### Cellular uptake and endocytic mechanism of coumarin-6 (C-6) loaded LPNs on Caco-2 cells

Caco-2 cells (1 × 10^5^ cells/well) were seeded in the 24 well plate overnight and incubated with coumarin-6 loaded LPN formulations at pH 7.4 and 6.5 for 6 h. After treatments, the cells were washed, detached, and then collected. The cells were centrifuged and suspended in cold PBS. The samples were analyzed by FACSCalibur flow cytometer (BD Biosciences, San Jose, CA, USA). Fluorescence was detected through a FL-1 filter for coumarin-6 and fluorescence signals were represented on a logarithmic scale. Data acquisition and analysis were calculated by BD FACStation™ software (BD Biosciences). Each group was analyzed in triplicate.

Endocytic mechanism of C-6/LPNs on Caco-2 cells was determined by pre-treating cells with different endocytosis inhibitors including poly-lysine (P-L; 10 µM, positive charge inhibitor), chlorpromazine (CPZ; 10 µM, clathrin-mediated endocytosis inhibitor), 5-(*N*,*N*-dimethyl) amiloride (DMA; 10 µg/ml, micropinocytosis inhibitor), nystatin (NYS; 20 µg/ml, and caveolae-mediated endocytosis inhibitor) for 60 min. The cells were then treated with coumarin-6 loaded nanoparticles for 6 h. After treatments, the harvested cells were centrifuged at 300*g* at 4 °C and then suspended in cold PBS. The samples were analyzed by FACSCalibur flow cytometer (BD Biosciences).

### Coumarin-6/LPN and miR-FAM/LPN intracellular localization

Caco-2 cells (2 × 10^5^ cells/well) were seeded in the 6 well plate. Coumarin-6/LPN and FAM-miR139/LPN-HR were added to the plate, treated for 1 or 6 h, respectively. The medium was removed and the cells were stained with LysoRed at 37 °C. After staining, cells were washed with PBS, and fixed with 4% paraformaldehyde at room temperature. Cells were permeabilized by Triton-X at room temperature. DAPI was used to stain cell nuclei at 37 °C. After mounting the samples, the cells were visualized by CLSM (Olympus FV10i, Olympus America Inc., Center Valley, PA, USA).

### Annexin V/PI staining

Caco-2 cells were seeded in a 6-well plate. Different treatments were added into the plate and incubated for 72 h. After treatments, the cells were collected, centrifuged at 300*g* at 4 °C for 8 min twice, and then suspended in PBS. Cells were stained by Annexin V-FITC Apoptosis Detection Kit (Strong Biotech Corporation, Taipei, Taiwan) in the dark at room temperature. The samples were analyzed by FACSCalibur flow cytometer.

### Cell cycle assay

Caco-2 cells (2 × 10^5^ cells/well) were seeded in a 6-well plate. Different treatments were added into the plate and incubated for 72 h. After treatments, the cells were harvested, centrifuged, and then suspended in PBS. Cells were fixed by 70% ethanol and stored in − 20 °C overnight. After that, cells were washed by PBS and centrifuged at 4 °C and re-suspended in cold PBS. Cells were stained by PI solution in the dark at room temperature. The samples were analyzed by FACSCalibur flow cytometer.

### Cell migration assay

Cell migration ability was measured using the Transwell inserts (Greiner, Frickenhausen, Germany). 2 × 10^5^ cells/insert were seeded in the upper chamber and incubated with different treatments for 48 h. After treatment, the medium on the upside chambers was removed carefully and fixed with 70% ethanol for 10 min at room temperature. Cells that remained on the upside of insert membrane were removed by cotton swabs. Inserts were immersed into 0.2% crystal violet and stained for 30 min at room temperature. Cells in five randomly selected visual fields (magnification, ×40) were counted in each Transwell chamber. The relative migration % was calculated by counting the number of cells that had migrated to the lower side of the membrane compared to the control group.

### Western blot assay

Caco-2 cells (2 × 10^5^ cells/dish) were seeded in the 6 cm dish, and various treatments were added and cultured for 72 h. Cells were lysed by RIPA buffer (Cell Signaling, Beverly, MA, USA) and protein was extracted. Protein quantification was determined by BCA TM Protein Assay Kit (Thermo Fisher Scientific, Waltham, MA, USA). Proteins were separated by sodium dodecyl sulfate-polyacrylamide gel electrophoresis (SDS-PAGE) and transferred to a polyvinylidene difluoride membrane. After blocking, the membranes were immersed in primary antibodies at 4 °C overnight, followed by incubation with secondary antibodies at room temperature for 1 h. The relative protein expression was detected by chemiluminescent imaging system (ImageQuant LAS 4000).

### Statistical analysis

Experimental data were expressed as the mean ± standard deviation (SD) and analyzed by Student’s t-test. P < 0.05 was considered statistically significant.

## Additional file

**Additional file 1: Figure S1.** Mass spectra of peptides and lipids. Mass spectra of (A) DSPE-PEG-NHS (B) peptide H (C) peptide R. Mass spectra of peptides and lipids were characterized by Matrix-Assisted Laser Desorption/Ionization Time-Of-Flight Mass Spectrometry (MALDI TOF MS).

## Data Availability

All data and materials are included in the manuscript.
