# Supplementary material for: Improving the anticancer effect of afatinib and microRNA by using lipid polymeric nanoparticles conjugated with dual pH-responsive and targeting peptides
Source: J Nanobiotechnology. 2019 Aug 19;17:89. doi: 10.1186/s12951-019-0519-6 (PMC6699136; doi:10.1186/s12951-019-0519-6)
Supplement: Supplementary file 1 — Additional file 1: Figure S1. Mass spectra of peptides and lipids. Mass spectra of (A) DSPE-PEG-NHS (B) peptide H (C) peptide R. Mass spectra of peptides and lipids were characterized by Matrix-Assisted Laser Desorption/Ionization Time-Of-Flight Mass Spectrometry (MALDI TOF MS). [file 12951_2019_519_MOESM1_ESM.docx]

**Additional figures**

**Improving the anticancer effect of afatinib and microRNA by using lipid polymeric nanoparticles conjugated with dual pH-responsive and targeting peptides**

Shu-Ting Hong^1,†^, Huaching Lin^2,†^, Chen-Shen Wang^1^, Chih-Hsien Chang^1^, Anya Maan-Yuh Lin^1,3,5^, James Chih-Hsin Yang^6^ and Yu-Li Lo^1,3,4,^*

*****Correspondence: yulilo@ym.edu.tw

^†^ Shu-Ting Hong and Huaching Lin contributed equally to this work

^1^Institute of Pharmacology, National Yang-Ming University, Taipei 112, Taiwan

^2^Cheng Hsin General Hospital, Taipei, Taiwan

^3^Faculty of Pharmacy, National Yang-Ming University, Taipei 112, Taiwan

^4^Center for Advanced Pharmaceutics and Drug Delivery Research, National Yang-Ming University, Taipei 112, Taiwan

^5^Department of Medical Research, Taipei Veterans General Hospital, Taipei 112, Taiwan

^6^Institute of Oncology, National Taiwan University, Taipei 106, Taiwan


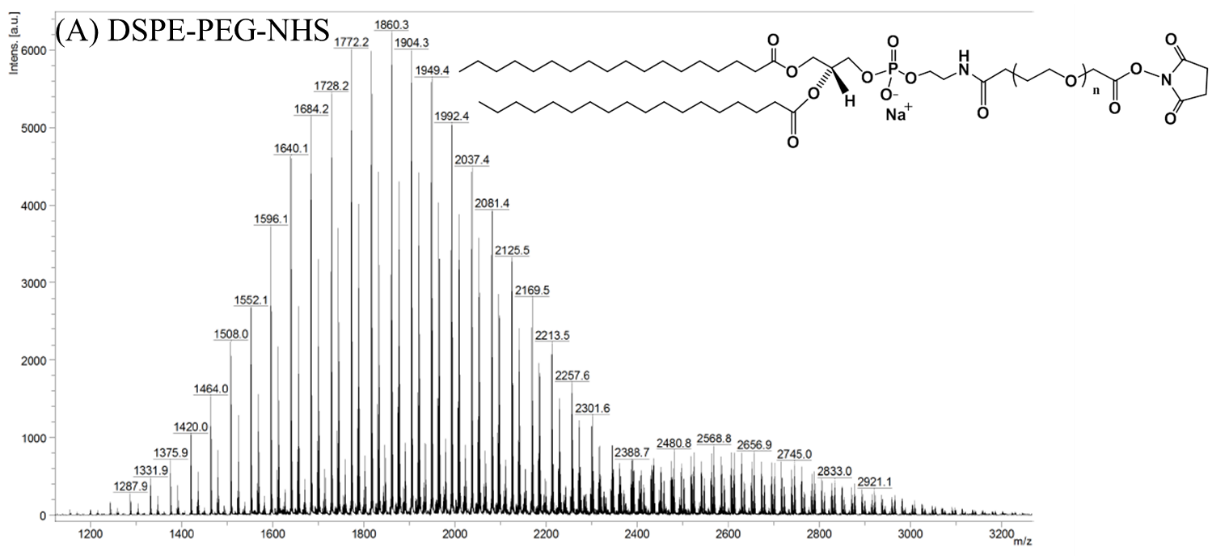


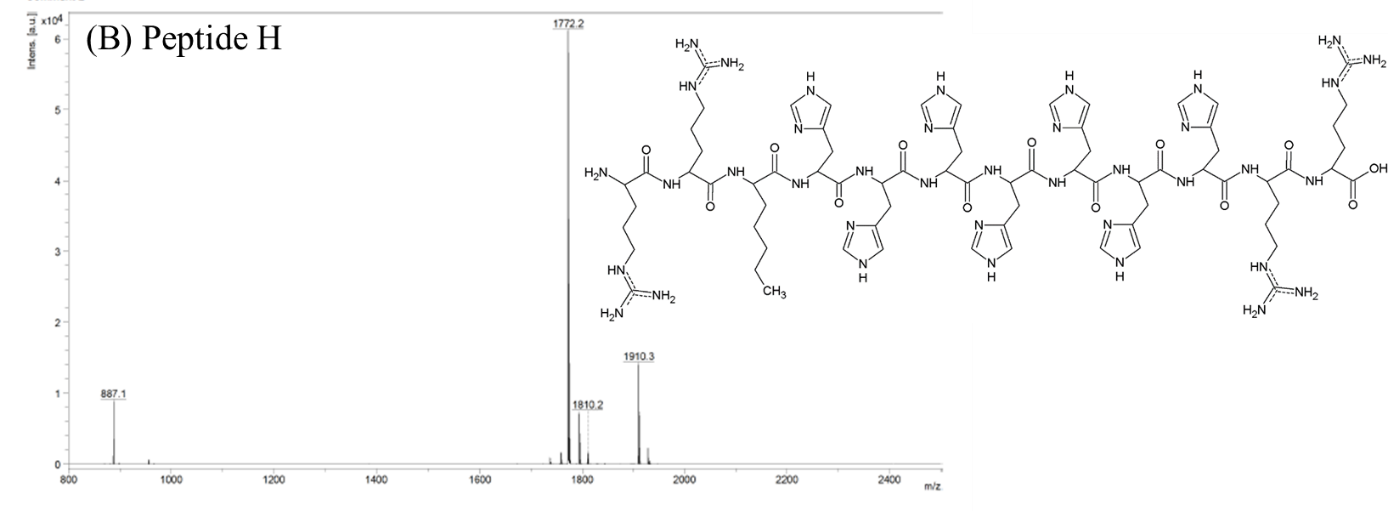


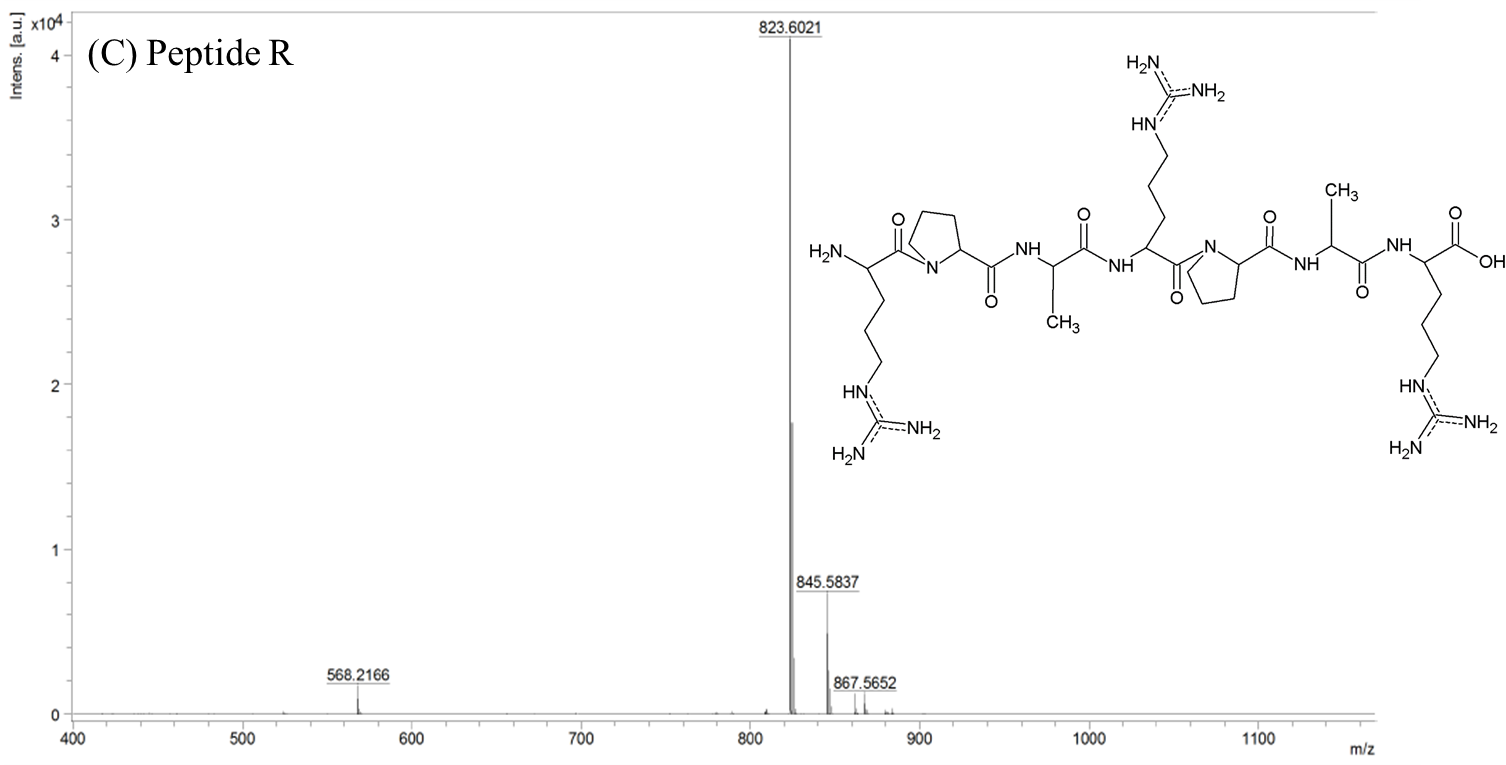


**Figure S1** Mass spectra of peptides and lipids. Mass spectra of (A) DSPE-PEG-NHS (B) peptide H (C) peptide R. Mass spectra of peptides and lipids were characterized by Matrix-Assisted Laser Desorption/Ionization Time-Of-Flight Mass Spectrometry (MALDI TOF MS).
